# Polynucleotide phosphorylase promotes the stability and function of Hfq-binding sRNAs by degrading target mRNA-derived fragments

**DOI:** 10.1093/nar/gkz616

**Published:** 2019-07-22

**Authors:** Todd A Cameron, Lisa M Matz, Dhriti Sinha, Nicholas R De Lay

**Affiliations:** 1 Department of Microbiology and Molecular Genetics, McGovern Medical School, The University of Texas Health Science Center, Houston, TX 77030, USA; 2 MD Anderson Cancer Center UTHealth Graduate School of Biomedical Sciences, The University of Texas Health Science Center, Houston, TX 77030, USA

## Abstract

In many Gram-negative and some Gram-positive bacteria, small regulatory RNAs (sRNAs) that bind the RNA chaperone Hfq have a pivotal role in modulating virulence, stress responses, metabolism and biofilm formation. These sRNAs recognize transcripts through base-pairing, and sRNA–mRNA annealing consequently alters the translation and/or stability of transcripts leading to changes in gene expression. We have previously found that the highly conserved 3′-to-5′ exoribonuclease polynucleotide phosphorylase (PNPase) has an indispensable role in paradoxically stabilizing Hfq-bound sRNAs and promoting their function in gene regulation in *Escherichia coli*. Here, we report that PNPase contributes to the degradation of specific short mRNA fragments, the majority of which bind Hfq and are derived from targets of sRNAs. Specifically, we found that these mRNA-derived fragments accumulate in the absence of PNPase or its exoribonuclease activity and interact with PNPase. Additionally, we show that mutations in *hfq* or in the seed pairing region of some sRNAs eliminated the requirement of PNPase for their stability. Altogether, our results are consistent with a model that PNPase degrades mRNA-derived fragments that could otherwise deplete cells of Hfq-binding sRNAs through pairing-mediated decay.

## INTRODUCTION

Small regulatory RNAs (sRNAs) are crucial modulators of cellular function and homeostasis in bacteria. Most sRNAs range in size from 50 to 250 nucleotides and can regulate the expression of hundreds of genes in response to specific signals (1). Typically non-coding sRNAs function by binding to and modifying the behavior of regulatory proteins or by directly base-pairing with RNA targets to modulate transcript stability and/or translation (2). Although sRNAs were classically characterized for their roles in facilitating bacterial stress response, subsequent research found that sRNAs control a myriad of physiological processes including biofilm formation (3,4), metabolism (5,6), motility (7,8) and virulence (9,10).

In *Escherichia coli* and other Gram-negative bacteria, one of the largest and most well-studied classes of sRNAs are those dependent on the sRNA chaperone protein Hfq. In addition to binding and protecting unpaired sRNAs from degradation, Hfq binds to mRNA targets and facilitates annealing between sRNA and mRNA (11–15). The torus-shaped Hfq hexamer has three RNA-binding surfaces, one on the rim and one each on the opposing distal and proximal faces. Each face of Hfq has affinity for distinct RNA motifs: the proximal face binds to poly-U rich sequences (16,17), the distal face binds to sequences containing an (AAN)_n_ motif (18,19), and the rim binds to AU-rich sequences (20–22). Hfq-dependent sRNAs are further categorized according to which faces these riboregulators interact with. Class I sRNAs bind the rim and proximal face of Hfq and their mRNA targets bind the distal face; in contrast, Class II sRNAs bind the distal and proximal faces and their mRNA targets bind the rim (23). Therefore, in order for an sRNA to regulate a particular mRNA sequence, the mRNA must possess both a base-pairing region at least partially complementary to the sRNA as well as the Hfq-binding motif that is not utilized by its cognate sRNA.

Pairing between an Hfq-dependent sRNA and its target can result in a variety of regulatory outcomes. In some cases, pairing remodels the 5′ UTR secondary structure so that the ribosome binding site (RBS) of the target mRNA is more easily accessible to the ribosome, resulting in positive regulation (24). However, sRNA–mRNA pairing more often leads to negative regulation by blocking the ribosomal 30S subunit from accessing the RBS and/or by destabilizing the target transcript (25–28). Decay of an sRNA–mRNA pair is typically initiated via an endoribonucleolytic cleavage by RNase E and is finalized by exoribonucleases that further degrade the resulting RNA fragments.

It was recently shown that the 3′-to-5′ phosphorolytic exoribonuclease PNPase is also important for Hfq-dependent sRNA function. Deletion of the gene encoding PNPase (*pnp*) paradoxically destabilizes sRNAs and reduces sRNA-mediated gene regulation, but the exact mechanism of this role has not yet been determined (29–32). PNPase has many other functions in the cell including roles in processing structured mRNA, rRNA, and tRNA and also performs general mRNA decay both independent of and in conjunction with RNase E (33). Although PNPase is a potent RNase that can degrade free sRNAs in vitro, it also forms a stable complex with Hfq and sRNAs together suggesting that the PNPase–sRNA–Hfq complex could have a role in facilitating sRNA-mediated gene regulation (31).

In this article, we present new data that provides insights into the molecular mechanism by which PNPase promotes sRNA stability and function. We show that loss of PNPase results in up-regulation of more than one hundred small mRNA-derived fragments and provide evidence that PNPase is directly responsible for their degradation. Many of these mRNA-derived fragments bind Hfq and originate from transcripts that base-pair with sRNAs, indicating that they are potentially generated by and capable of sRNA–mRNA base-pairing. Finally, we demonstrate that disruption of either target mRNA binding to Hfq or sRNA–mRNA pairing results in suppression of sRNA instability in the absence of PNPase. Based on these results, we suggest a new model wherein PNPase facilitates sRNA stability and function by actively degrading mRNA fragments, some of which would otherwise associate with Hfq and base-pair with additional sRNA molecules leading to sRNA depletion.

## MATERIALS AND METHODS

### Bacterial strains and growth conditions

All strains and plasmids used in this study are derivatives of *E. coli* K-12 strain MG1655 (*rph-1*) and are listed in the Supplementary Table S1. Primers and probes used for strain construction are listed in the Supplementary Table S2. Strain construction is described in the Supplementary Materials and Methods. Strains were grown in liquid medium or agar plates containing Lennox lysogeny broth (LB) supplemented with antibiotics (25 μg/ml kanamycin; 25 μg/ml chloramphenicol; 100 μg/ml ampicillin) when appropriate and grown aerobically at 37°C. Overnight cultures were diluted 1:200 fold in LB medium and grown until desired densities were reached. Growth was determined by measuring the optical densities of liquid cultures at 600 nm (OD_600_).

### RNA sample collection

Overnight cultures were diluted into fresh LB and grown with shaking to the indicated OD_600_ before collecting samples. Where indicated, RyhB or CyaR was first induced for 15 min prior to initial collection; RyhB was induced by addition of 2,2′-dipyridyl (Sigma) to cultures at a final concentration of 250 μM and CyaR was induced by addition of cAMP (Sigma) to a final concentration of 5 mM. For RNA stability curves, rifampicin (Fisher Scientific) was added at 16 min post-induction to a final concentration of 250 μg/ml, then additional samples were collected at either 1, 2, 4 and 6 min or 2, 5, 10 and 15 min after rifampicin addition.

### RNA extraction

Samples of 700 μl were withdrawn directly from cultures and collected into 2 ml tubes containing 100 μl of 8× lysis buffer (92 μl dH_2_O, 160 μl 3M NaOAc, 1200 μl 10% SDS, 48 μl 0.5 M EDTA) and 800 μl of 5:1 phenol:chloroform (pH 4.1, Fisher Scientific) pre-warmed to 65°C. Samples were processed at 65°C for five min with intermittent shaking, then centrifuged at 4°C. The aqueous layer was transferred to a new Eppendorf tube and extracted once or twice with 25:24:1 phenol:chloroform:isoamyl alcohol (pH 6.7, Fisher Scientific) or with pure chloroform (Fisher Scientific). Total RNA was alcohol-precipitated from the final aqueous fraction and suspended in Tris-EDTA (TE) buffer or DEPC-treated water. The final RNA concentration was quantified using a Nano Drop 2000 spectrophotometer (Thermo Fisher Scientific).

### Immunoprecipitations

For Hfq immunoprecipitations, overnight cultures were diluted into 25 ml of fresh LB medium and grown to an OD_600_ of 1.0. Cells were pelleted, washed, and frozen as described previously (31). Immunoprecipitations were performed as previously described (12) using anti-Hfq antiserum obtained from Dr Susan Gottesman (NCI). For PNPase immunoprecipitations, overnight cultures were diluted into 60 ml of fresh LB medium and grown with shaking. Upon reaching an OD_600_ of 0.85, RyhB was induced for 15 min by addition of 2,2′-dipyridyl to cultures at a final concentration of 250 μM. Cultures were collected on ice after 15 min at a final OD_600_ of 0.96–1.04. All further manipulations were performed at 4°C. Cultures were pelleted and washed twice with Tris-buffered saline (TBS; 50 mM Tris, 150 mM NaCl, pH of 7.4). Pellets were flash-frozen, then suspended in 500 μl of TBS containing 5 μl of HALT protease inhibitor (Thermo Fisher Scientific) and 2 μl of Superase RNase inhibitor (Ambion) and mixed with an equal volume of 0.1 mm glass beads. Samples were vortexed for 10 min, alternating between vortexing and ice incubation every 30 s. After addition of 500 μl of TBS, each sample was vortexed for an additional 30 s followed by centrifugation for 30 min at 18 000 × g. The soluble fraction was transferred to a new tube, and TBS plus 5 μl of Superase was added to bring the volume to 1.0 ml. Of this, 50 μl was retained as the input fraction. The remaining sample was incubated with 75 μl of anti-FLAG M2 agarose resin for 2 h with mixing. Resin was washed three times with 1.5 ml of TBS, and bound proteins were subsequently eluted by incubation with 150 μg/ml of 3xFLAG peptide (Millipore Sigma) in 250 μl of TBS for 30 min with mixing. Samples were pelleted after the addition of 250 μl of TBS, and the supernatant was retained as the output fraction. RNA was isolated from input and output fractions by hot phenol/chloroform extraction as described above.

### Northern blots

For polyacrylamide gels, 3 μg of each RNA sample was prepared with formamide loading buffer and loaded on 10% polyacrylamide Tris-borate-EDTA (TBE)-urea gels and electrophoresed at 60–85 V unless noted otherwise. The RNA was then transferred to a Zeta-Probe GT membrane (Bio-Rad) using a Trans-Blot SD semidry transfer apparatus (Bio-rad) following the manufacturer's guidelines. For agarose gels, 10 μg of each sample was prepared with formaldehyde and formamide loading buffer and loaded on 1.2% agarose 3-(*N*-morpholino)propanesulfonic (MOPS) gels. Gels were electrophoresed at 65 V and transferred to Zeta-Probe GT membrane by overnight capillary transfer. After transfer, membranes were UV-crosslinked and hybridized overnight with 100 ng/ml of 5′ biotinylated DNA probes (Supplementary Table S2) in ULTRAhyb (Ambion) hybridization buffer at 42°C. Blots were developed using the BrightStar BioDetect kit protocol (Ambion).

### Analysis of blots

Blots were imaged with a ChemiDoc MP imager (Bio-Rad) and quantified using Image Lab (Bio-Rad). Data analysis and half-life calculations of RNA stability time course experiments were conducted using R (R Foundation for Statistical Computing, Vienna, Austria https://www.R-project.org) and visualized using the ggplot2 package (https://ggplot2.tidyverse.org). To plot decay curves for each sample, the average intensity of each replicate time course was normalized, and the individual time points were each plotted as a fraction of the average normalized *T*_0_ value. Half-lives and standard error were derived from the best-fit exponential decay curves of the combined replicate data.

### Library preparation and RNA sequencing

Total RNA samples collected from cultures at an OD_600_ of 1.0 were subjected to DNase treatment (DNase Turbo; Ambion) following the manufacturer's protocol. Sample mixtures (total reaction volume of 100 μl) were incubated for 1 h at 37°C and the reaction was stopped by addition of 100 μl of DEPC-treated water and 200 μl of neutral phenol–chloroform–isoamyl alcohol (Fisher Scientific). DNase-treated RNA samples were phenol extracted, alcohol precipitated, and RNA concentration was measured. 5 μg of DNase-treated RNA was subjected to ribosomal RNA removal (RiboZero™ rRNA Removal for Gram-negative Bacteria, Illumina). For mRNA sequencing, libraries were constructed by Macrogen (Rockville, MD) from the rRNA-depleted samples using the Illumina TruSeq Stranded Total RNA library kit. For short RNA sequencing of total RNA and RNA immunoprecipitated with Hfq, rRNA-depleted samples were subjected to RNA fragmentation using the Ambion RNA fragmentation kit (AM8740) followed by RNA 5′ polyphosphatase treatment (Epicenter), which was performed to facilitate 5′ adapter ligation. Libraries were generated by Macrogen (Rockville, MD, USA) using the TruSeq Small RNA library kit (Illumina). All high throughput RNA sequencing was performed using 100 bp paired end read sequencing with an Illumina HiSeq2000 sequencer.

### RNA sequencing analysis

High throughput RNA sequencing data were preprocessed for alignment with Cutadapt (34) to remove Illumina sequencing adapters and low-quality bases (Phred < 20) from the ends of reads. Trimmed read pairs were then aligned to the NCBI RefSeq NC_000913.3 for *E. coli* MG1655 using BWA-MEM (35), and the resulting SAM files were converted to BAM files using SAMtools (36). Aligned reads were assigned to individual genes and quantified using the annotated genome using featureCounts (37), and differential expression analysis among samples was performed with DESeq2 (38). Read coverage was calculated and graphed using a custom R script (RCoverage, available at https://github.com/ta-cameron/RCoverage). These analyses were performed in part using high-performance computing resources of the Texas Advanced Computing Center (TACC) at The University of Texas at Austin.

Up-regulated mRNA fragments were detected using a custom analysis in R. In brief, read coverage of the WT and Δ*pnp* replicates was first calculated genome-wide over 5 nt intervals, then analyzed by DESeq2 to identify up-regulated 5 nt segments with adjusted *P*-values <0.05. All contiguous significant segments totaling 60 nt or greater were combined, resulting in 463 significantly up-regulated fragments. *Z*-scores were calculated for each fragment by comparing in the Δ*pnp* strain expression of the fragment versus expression within flanking 350 nt regions extending 150–500 nt from the fragment on each side. Z-scores were also calculated for any overlapping genes, if present. To reduce the initial fragment hits to those most likely to be biologically relevant, the initial fragment hits were further filtered for a minimum log_2_ fold change of 1.5, average Δ*pnp* coverage of at least 300, and the smallest expression *Z*-score >1, resulting in 106 distinctly expressed fragments. Fragments were annotated relative to genes and the furthest known operon boundaries (39,40); operons lacking data were approximated by extending 75 nt from the beginning of the first gene and the end of the last gene. Fragments further than 75 nt from an operon boundary were considered intergenic regions (IGR). Fragments overlapping within 20 nt of the start codon were considered to overlap the RBS and fragments otherwise wholly within genes were assigned to CDS. All other fragments within operons were assigned as 5′ UTR or 3′ UTR relative to the nearest gene in the operon. For comparison with RIL-seq sRNA–mRNA hits, each fragment was compared with each reported RIL-seq sRNA–mRNA interaction to identify fragments of mRNAs that are known targets of sRNAs. For evaluation of RIL-seq hit enrichment, 10,000 fragments with lengths and compositions identical to the detected fragments were randomly generated among all *E. coli* MG1655 genes not associated with the detected fragments, which were then annotated and evaluated as described above.

## RESULTS

### mRNA-derived fragments accumulate in the absence of PNPase

**Figure 1.**
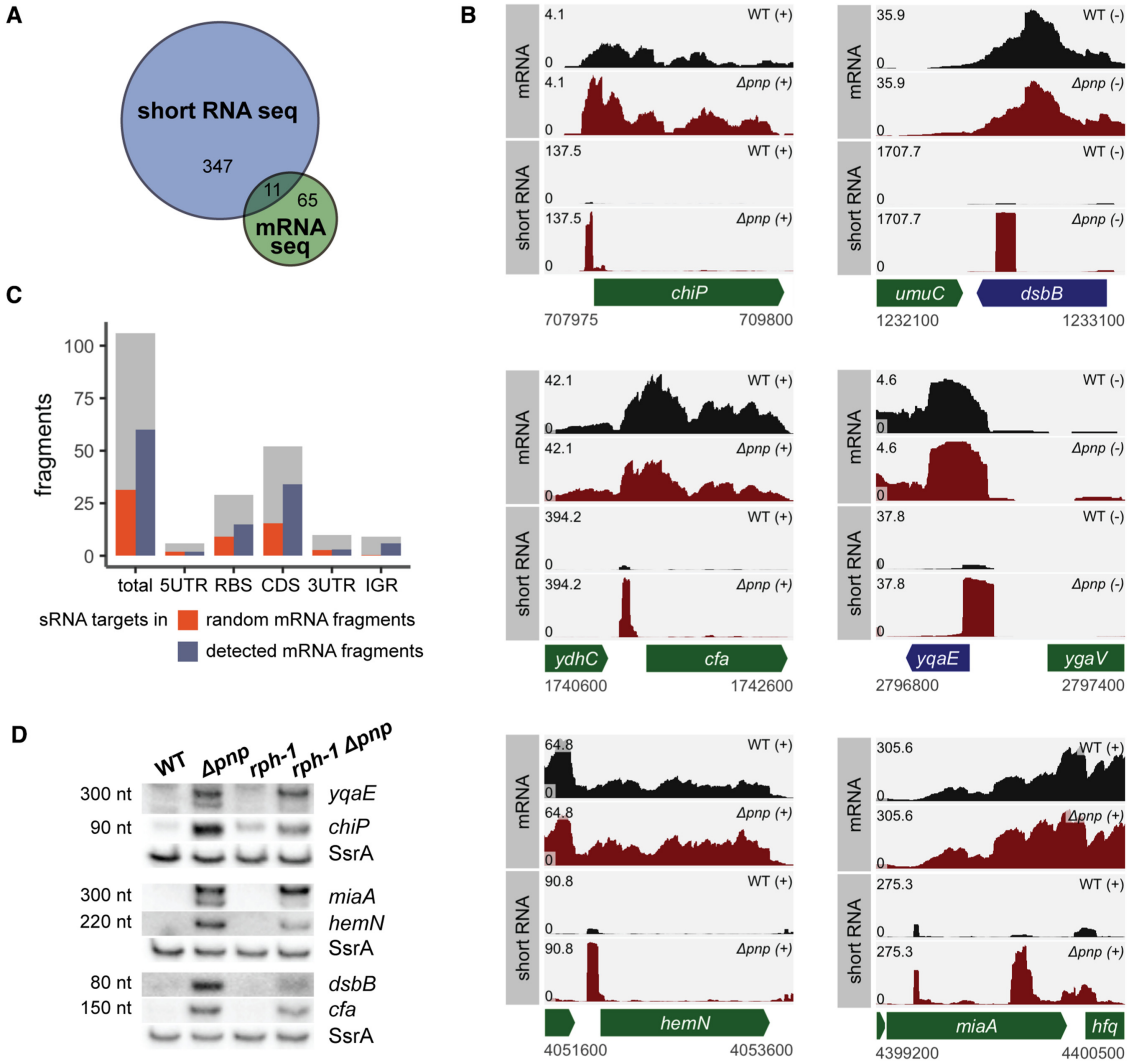
PNPase is critical for the decay of specific mRNA-derived fragments. (**A**) A Venn diagram indicating the number of common and uniquely identified differentially regulated genes between the short RNA-seq and mRNA-seq data sets. Numbers represent genes identified by DESeq2 as significantly differentially expressed with a log_2_ fold-change in expression of at least 1.5 when comparing the WT (KR10000) and Δ*pnp* (NRD999) strains. (**B**) RNA-seq read coverage of mRNA and short RNA library preparations of WT (KR10000; black) and Δ*pnp* (NRD999; red) strains. In the absence of PNPase, numerous short RNA fragments accumulate, including those corresponding to the *chiP* RBS, *dsbB* CDS, *cfa* 5′ UTR, *yqaE* RBS, *hemN* 5′ UTR, and *miaA* CDS. Coverage represents depth per million paired-end fragments and was averaged between two normalized replicates. (**C**) 106 short RNA fragments accumulating in the Δ*pnp* strain were computationally identified. Gray bars indicate the number of RNA fragments corresponding to each gene feature type (5′ UTR, RBS, CDS, 3′ UTR, IGR). Blue bars represent the number of identified fragments that are associated with RNAs shown to interact with Hfq-binding sRNAs in prior RIL-seq experiments (41). Orange bars indicate how many RNA fragments would be expected to associate with mRNAs previously shown to interact with Hfq-binding sRNAs in RIL-seq experiments if RNA fragments were chosen at random from the *E. coli* K12-MG1655 transcriptome. (**D**) Northern blots probed for the presence of the RNA fragments shown in B in the wild-type (KR10000), Δ*pnp* (NRD999), *rph-1* (NRD1138), and *rph-1* Δ*pnp* (NRD1139) strains grown to OD_600_ 1.0. SsrA served as a loading control. An expanded view of the northern blots is shown in Supplementary Figure S1.

To elucidate the mechanism by which PNPase stabilizes sRNAs, we first examined the impact of deletion of *pnp*, the gene encoding PNPase, on *E. coli* gene expression by performing high throughput RNA sequencing of mRNAs isolated from the wild-type *E. coli* K-12 MG1655 strain KR10000 (*rph*^+^) or a derived Δ*pnp* strain (NRD999) grown to late log phase (OD_600_ of 1.0). Comparison of the transcriptome of the wild-type and the derived Δ*pnp* strain identified 76 genes that were differentially expressed between these strains, i.e. 15 genes were significantly down-regulated and 61 genes were significantly up-regulated in the Δ*pnp* strain relative to the parental strain (Figure 1A, Supplementary Supplementary Table S3); genes were considered statistically significant if they had a log_2_ fold change of at least 1.5 and a false discovery rate (FDR) adjusted *P*-value <0.05. Approximately 25% of those genes that are significantly altered in expression upon deletion of *pnp* encode mRNAs that have previously been shown to be regulated by Hfq-dependent sRNAs.

In preparing the mRNA-seq libraries, most sequences shorter than 150 nt were eliminated by the Illumina TruSeq Stranded Total RNA library kit protocol. In order to assess the impact of the *pnp* deletion on the levels of smaller transcripts including mRNA fragments and most sRNAs involved in Hfq-mediated gene regulation, an alternative short RNA-seq library preparation was employed. In this protocol, rRNA-depleted RNA samples were fragmented to a relatively uniform size by metal ion hydrolysis prior to library construction using the Illumina TruSeq Small RNA library kit. With this approach, subsequently referred to as ‘short RNA-seq’, comparison of the transcriptomes of the wild-type and the derived Δ*pnp* strains identified 358 genes that were significantly differentially expressed (194 up-regulated and 164 down-regulated transcripts), most of which had not been identified in the initial mRNA-seq analysis (Figure 1A, Supplementary Table S3).

Subsequent analysis further revealed numerous short RNA fragments ∼75–150 nt in length that were highly up-regulated in the absence of PNPase (Figure 1B, Supplementary Figure S1). A systematic global search was conducted to identify short transcripts enriched in the absence of PNPase. Of the 106 RNA regions identified by this analysis, nearly all were small fragments of known or predicted transcripts with the vast majority (>75%) originating wholly from within the CDS or overlapping the RBS of a protein-coding gene (Figure 1C, gray bars). These RNA fragments were then cross-referenced with previously published RIL-seq data that designated putative sRNA targets genome-wide (41), and approximately 57% of the RNA fragments were derived from transcripts previously identified as sRNA targets (Figure 1C, blue bars). This is 2-times higher than the discovery rate of RIL-seq hits found among randomly selected fragments of the same composition (Figure 1C, orange bars) suggesting that the RNA fragments identified in our analysis were indeed enriched for those with potential sRNA interactions. Among the mRNA-derived fragments that were enriched with potential sRNA interactions were fragments containing the *yqaE* RBS, *chiP* RBS, *miaA* 3′ UTR, *hemN* 5′ UTR, *dsbB* coding sequence (CDS) and *cfa* 5′ UTR (Figure 1B).

To validate these RNA-seq results, we first tested whether we could detect these fragments by northern blot analysis in total RNA samples isolated from the wild-type *E. coli* strain and Δ*pnp* mutant. The *yqaE* RBS, *chiP* RBS, *miaA* 3′ UTR, *hemN* 5′ UTR, *dsbB* CDS and *cfa* 5′ UTR fragments were readily detected at the predicted sizes based upon the RNA-seq analysis (Figure 1D). While these mRNA-derived fragments accumulated in the Δ*pnp* strain, the corresponding full-length mRNA was not substantially increased in this strain as determined by mRNA-seq (Figure 1B) or northern blot analyses (Supplementary Figure S1B). For three fragments, *yqaE* RBS, *miaA* 3′ UTR and *cfa* 5′ UTR, additional intermediate-length transcripts were also detected in the Δ*pnp* strains by northern blot (Supplementary Figure S1B). Of all 106 fragments, only one, a fragment from the 3′ UTR of *pnp* and likely an artifact of the *pnp* deletion, was also identified as significantly differentially expressed in the mRNA-seq results (Supplementary Tables S3 and S4). Together these results suggest that efficient decay of these mRNA fragments, most of which are produced from transcripts regulated by Hfq-binding sRNAs, requires the presence of PNPase.

Next we investigated whether these fragments were affected by mutations in other proteins with roles in sRNA-mediated gene regulation (RNase PH, RNase E and Hfq). Previously we demonstrated a synergistic decrease in sRNA stability by the *pnp* deletion and a null mutation in *rph* (*rph-1*), the gene encoding RNase PH (32). Although the two homologous proteins share a common catalytic core domain, RNase PH lacks the RNA-binding KH and S1 domains of PNPase and has a more limited impact on sRNA stability compared to PNPase (32). We examined whether the *rph-1* mutation impacted the levels of these fragments, and as shown in Figure 1D, introduction of the *rph-1* allele had a minor impact on the levels of most of the mRNA-derived fragments. Likewise, a C-terminal truncation in RNase E (*rne-131*), which removes portions of the protein that interact with PNPase and Hfq, did not impact the generation of most fragments (Supplementary Figure S1A). However, deletion of Hfq eliminated, decreased, or altered the sizes of bands observed for the *yqaE* RBS, *chiP* RBS, *miaA* 3′ UTR and *cfa* 5′ UTR fragments, suggesting a role for Hfq in either their generation or stabilization (Supplementary Figure S1A).

**Figure 2.**
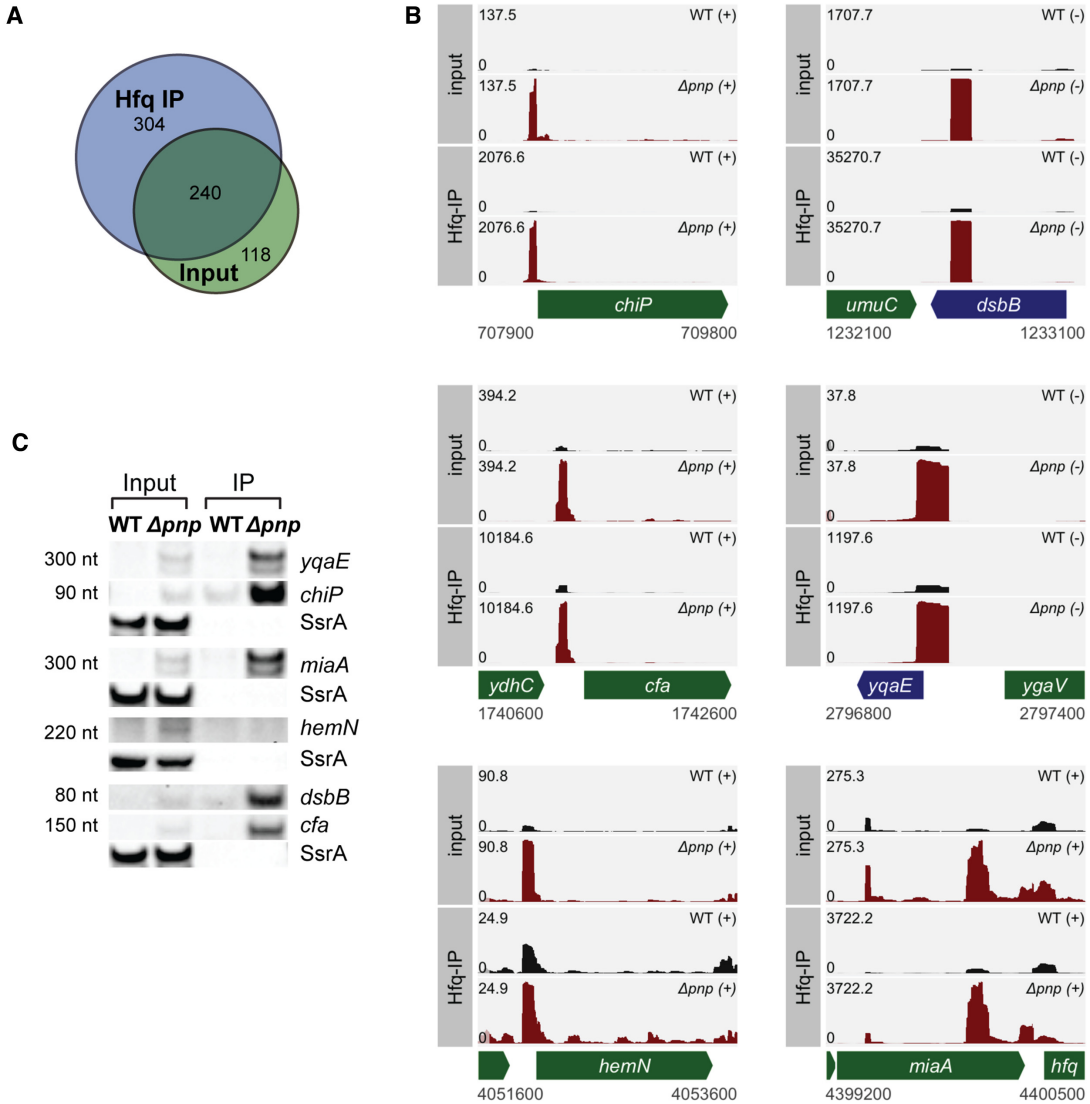
mRNA-derived fragments that accumulate in the absence of PNPase interact with Hfq. (**A**) A Venn diagram indicating the number of common and uniquely identified differentially regulated genes between the Hfq input and co-IP data sets. Numbers represent genes identified by DESeq2 as significantly differentially expressed with a log_2_ fold-change in expression of at least 1.5 when comparing the WT (KR10000) and Δ*pnp* (NRD999) strains. (**B**) RNA-seq read coverage of short RNA library preparations of input RNA and RNA co-immunoprecipitated with Hfq for the WT (KR10000; black) and Δ*pnp* (NRD999; red) strains for the same regions shown in Figure 1B. Coverage represents depth per million paired-end fragments and was averaged between two normalized replicates. (**C**) Input and Hfq co-immunoprecipitation fractions probed by northern blot for the presence of the RNA fragments shown in B in the wild-type (KR10000) and Δ*pnp* (NRD999) strains. SsrA served as a loading control. An expanded view of the northern blots is shown in Supplementary Figure S2.

In order to assess how a *pnp* deletion impacts the population of RNA that interacts with Hfq, short RNA-seq was also performed on Hfq co-immunoprecipitation (co-IP) fractions collected in parallel with the total RNA samples. Comparisons of the short RNA-seq total RNA and RNA that co-IPed with Hfq between the wild-type and Δ*pnp* strains indicated that most genes enriched in the total RNA samples for the Δ*pnp* strain were also highly enriched in the Hfq immunoprecipitant (Figure 2A). Of the 106 mRNA-derived fragments, 75 were present in the Hfq co-IP fraction as determined by visual inspection of read coverage (Supplementary Table S4) including the *yqaE, chiP, miaA, hemN, dsbB* and *cfa* fragments examined above (Figure 2B). Comparison between the normalized coverage depth of the input and Hfq co-IP fractions of the Δ*pnp* strain revealed that five of these six fragments (excluding *hemN*) were enriched by approximately 14- to 34-fold in the Hfq co-IP fraction. In total, 49 fragments were present at similar or increased levels in the Hfq co-IP, and 26 fragments were notably enriched (log_2_ fold change > 1.5) in the Hfq fraction in comparison to the input transcriptome. Moreover, the median Hfq co-IP enrichment of fragments whose sequence directly overlapped the target sequences obtained by RIL-seq was ∼4.3-fold over the input, far higher than for fragments not identified by RIL-seq either because the transcript was not identified as a target of an sRNA or the fragment was not within the precise boundaries of the sRNA-target sequence obtained by RIL-seq (Supplementary Figure S2A and Supplementary Table S4).

Next, we repeated the Hfq co-IPs and tested for the presence of *yqaE* RBS, *chiP* RBS, *miaA* 3′ UTR, *hemN* 5′ UTR, *dsbB* CDS and *cfa* 5′ UTR fragments by northern blot. As shown in Figure 2C, all of the RNA fragments except *hemN* were detected by northern blot in both the total RNA and Hfq co-IP fractions of the Δ*pnp* strain. Altogether, these results indicate that many of the mRNA-derived fragments up-regulated in a Δ*pnp* strain are derived from mRNAs targeted by sRNAs and bind Hfq.

### The enzymatic activity of PNPase is required for the degradation of these Hfq-binding mRNA-derived fragments and stabilization of Hfq-dependent sRNAs

**Figure 3.**
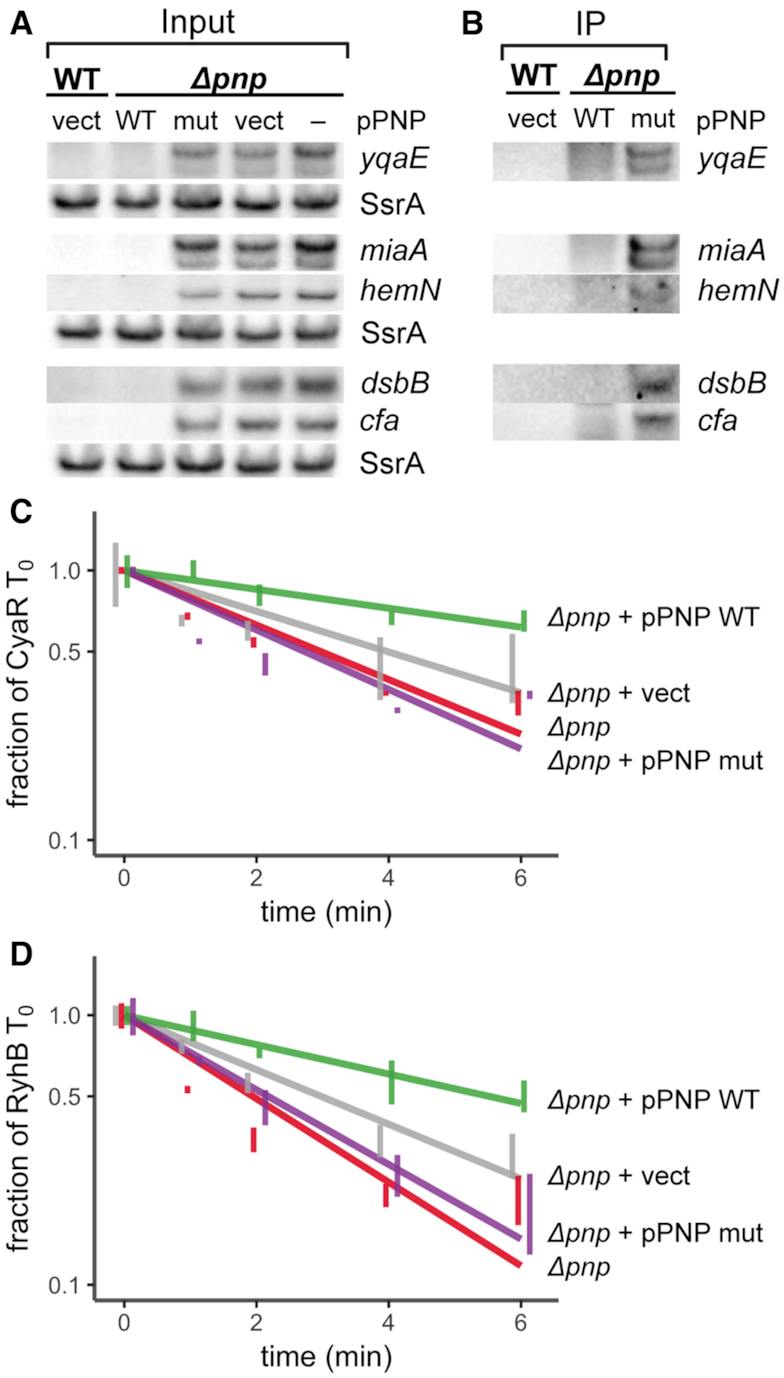
The active site of PNPase is required for decay of mRNA-derived fragments and for stabilization of sRNAs, but not for RNA binding. (**A**) Steady-state expression of RNA fragments in a WT (KR10000) strain containing the vector control (pTC396; WT vect) or in a Δ*pnp* strain (NRD999; Δ*pnp* –) containing pPNP expressing the PNPase-3x FLAG wild-type (pTC352; Δ*pnp* WT), a derived S438A catalytic mutant (pTC354; Δ*pnp* mut), or the vector control (pTC396; Δ*pnp* vect). Cultures were subjected to dipyridyl treatment for 15 min immediately prior to collection of samples at OD_600_ of 1.0. Samples were prepared for PNPase-3xFLAG pulldowns then total RNA was isolated from a small fraction of each sample as input RNA and probed the presence of the indicated RNA fragments. SsrA served as a loading control, with 4 μg total RNA loaded per lane. (**B**) Immunoprecipitations were subsequently performed using anti-FLAG antibody. RNA isolated from PNPase-3xFLAG pulldown fractions was probed for the presence of RNA fragments. Each lane contains RNA collected from an equal number of cells. (C, D) Stability curves of CyaR (**C**) and RyhB (**D**) with a *pnp* deletion strain or derived strains harboring an empty P*_lac_* based expression vector (pTC396; vect), a plasmid expressing PNPase (pTC352; WT), or a plasmid expressing the PNPase S438A mutant (pTC354; mut). Strains were inoculated in culture media containing 100 μM IPTG to induce expression of PNPase. Expression of each sRNA was induced in exponential phase cultures for 15 min by addition of dipyridyl. Total RNA was collected at 0, 1, 2, 4 and 6 min after addition of rifampicin to inhibit further transcription, and sRNA levels were assessed by northern blot. Lines indicate best-fit exponential decay curves of three replicates, and error bars indicate the standard error of each time point. SsrA served as a loading control. For C and D, the Δ*rph* Δ*pnp* strain TC292 carrying *cyaR* under the control of the *ryhB* promoter and the *rph-1* Δ*pnp* strain NRD1139 carrying *ryhB* under its native promoter were used. Expanded views and representative northern blots are shown in Supplementary Figure S3.

mRNA-derived fragments that showed increased abundance in the Δ*pnp* strain compared to the *pnp^+^* parental strain could be substrates degraded by PNPase or could be indirectly impacted by this protein. We postulated that if these mRNA-derived fragments are substrates of PNPase, then the 3′-to-5′ exoribonuclease activity of PNPase should be required for their decay and PNPase should interact with these mRNA-derived fragments. To specifically assess if the 3′-to-5′ exoribonuclease activity of PNPase plays a role in the degradation of these fragments, a plasmid expressing a 3x-FLAG-tagged form of either wild-type PNPase or a derived S438A catalytic mutant under control of an IPTG-inducible promoter was transformed into the Δ*pnp* background. Expression of wild-type PNPase and the derived catalytic mutant from the plasmid was optimized so that these proteins were expressed at levels comparable to PNPase expressed from its native chromosomal locus (Supplementary Figure S3A,B). As shown in Figure 3A, all five of the mRNA fragments examined (*yqaE* RBS, *miaA* 3′ UTR, *hemN* 5′ UTR, *dsbB* CDS, and *cfa* 5′ UTR) were generated in the presence of the PNPase catalytic mutant but not the wild-type protein indicating that catalytic activity of PNPase is required for the degradation of these RNAs. Next, we tested if these fragments interact with PNPase *in vivo* by assessing their ability to co-IP with the wild-type PNPase and the S438A catalytic mutant. Northern blots confirmed that the PNPase catalytic mutant pulled down each of these fragments (Figure 3B). However, none of the five mRNA fragments examined co-immunoprecipitated with wild-type PNPase. Overall these results suggest that the small mRNA-derived fragments up-regulated in the Δ*pnp* strain are substrates normally degraded by PNPase.

Previously, we found that multiple sRNAs are destabilized in a *pnp* deletion strain (8,31,32). Since many of the mRNA fragments identified above as up-regulated in the Δ*pnp* strain have sRNA pairing sites (Figure 1C), bind Hfq (Figure 2, Supplementary Figure S2), and are putative PNPase substrates, we hypothesized that the accumulation of these fragments in the absence of PNPase catalytic activity might result in increased degradation of sRNAs that pair with them. Thus, to determine if PNPase catalytic activity is important for sRNA stability, we assessed the decay of the sRNAs CyaR and RyhB by RNA stability assays in Δ*pnp* strains carrying plasmids expressing either wild-type PNPase-3xFLAG or a derived catalytically-inactive PNPase S438A mutant. As shown in Figure 3C and D, both sRNAs were stabilized by expression of the wild-type PNPase. In contrast, expression of the catalytically inactive mutant in the Δ*pnp* background did not restore sRNA stability. Together, these results indicate that catalytic activity of PNPase is the primary factor stabilizing these sRNAs.

### The role of Hfq in sRNA decay in the absence of PNPase

**Figure 4.**
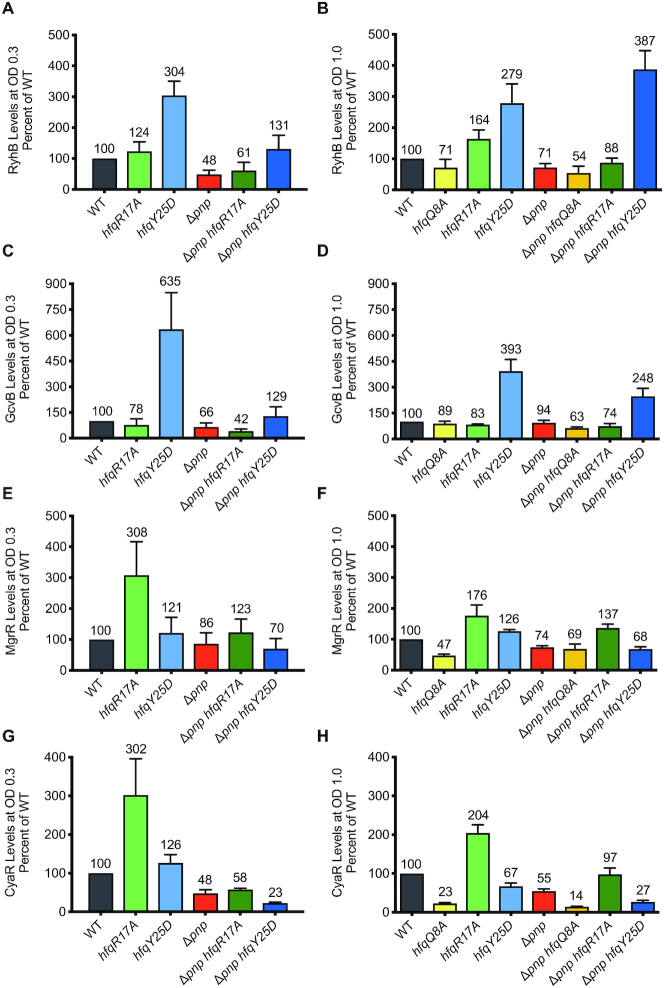
Substitutions in Hfq that block pairing of sRNAs with target mRNAs suppress the defect in sRNA levels that occurs in a Δ*pnp* strain. Overnight cultures of strain NRD1138 (WT *rph-1*) and derived strains carrying the *pnp* deletion (Δ*pnp*, DS070) and harboring mutations resulting in a substitution in a residue in the rim (R17A), distal face (Y25D), or proximal face (Q8A) of Hfq (*hfqR17A*, DS060; *hfqY25D*, NRD1410; *hfqQ8A*, DS058; Δ*pnp hfqR17A*, NRD1474; Δ*pnp hfqY25D*, NRD1478; Δ*pnp hfqQ8A*, NRD1473) were diluted into fresh LB medium and grown to early exponential phase (OD_600_ of 0.3) or late exponential phase (OD_600_ of 1.0). For early exponential phase cultures, RyhB and CyaR sRNAs were induced by the addition of 2,2′-dipyridyl or cAMP, respectively. Total RNA was extracted from cultures 15 min after induction and sRNA levels were examined by northern blot probing for RyhB (**A**), GcvB (**C**), MgrR (**E**), or CyaR (**G**) as described in Materials and Methods. For cultures grown to late exponential phase, total RNA was immediately extracted as described above from cultures for each strain after it reached an OD_600_ of 1.0. RyhB (**B**), GcvB (**D**), MgrR (**F**) and CyaR (**H**) were measured by northern blot analysis. sRNA levels were normalized to the control RNA SsrA, the level of each sRNA in WT was set to 100%, and sRNA levels were scaled relative to WT. The results presented in the bar graph represent the mean of three independent experiments and the error bars represent the standard error of the mean.

Protection of sRNAs by PNPase is limited to Hfq-dependent sRNAs (29–32,42,43) suggesting a role for Hfq in the destabilization of sRNAs in the absence of PNPase. Hfq is a canonical RNA chaperone that facilitates sRNA–mRNA target annealing (11,44,45). Two major classes of Hfq-dependent sRNAs have been described (21,23). Class I sRNAs, including RyhB and GcvB, interact with the proximal and rim surfaces of Hfq, and their mRNA targets bind the distal region. Conversely, Class II sRNAs, such as CyaR and MgrR, bind the proximal and distal regions of Hfq, and their cognate mRNAs interact with positively charged patches along the rim (21,23). We hypothesized that transcripts accumulating in the Δ*pnp* strain bind Hfq, which facilitates annealing to their cognate sRNAs resulting in sRNA decay. To test this hypothesis, we examined the effects of substituting a key residue of Hfq on the distal face or rim critical for the binding of Class I and Class II mRNA targets, respectively, on the stability of Class I and Class II sRNAs. Specifically, we predicted that introduction of a mutation in *hfq* resulting in the Y25D (*hfqY25D*) or R17A (*hfqR17A*) substitution into a Δ*pnp* strain would suppress the stability defect of Class I or Class II sRNAs, respectively. We first examined steady-state levels of seven sRNAs at early and late exponential phase (Figure 4, Supplementary Figure S4). In the *pnp*^+^ background, we observed that levels were highest for the Class I sRNAs RyhB, GcvB and ArcZ in the *hfqY25D* strain, whereas levels of the Class II sRNAs MgrR, CyaR, McaS and ChiX were highest in the *hfqR17A* strain. As expected, steady state levels were lower in Δ*pnp* relative to WT for the majority of sRNAs tested, except for GcvB at an OD_600_ of 1.0. Introduction of the *hfqY25D* allele into the Δ*pnp* strain completely suppressed the defect in expression of the Class I sRNA RyhB in the Δ*pnp* strain background during late exponential growth, but not during early exponential phase; however, for the other Class I sRNAs (GcvB and ArcZ), the *hfqY25D* allele was not able to eliminate the negative impact of the *pnp* deletion on steady state levels (Figure 4A-D, Supplementary Figure S4A and B). Conversely for Class II sRNAs, which interact with the distal face of Hfq, steady state levels in the Δ*pnp hfqY25D* strain were similar to or lower than those in Δ*pnp* alone (Figure 4E–H, Supplementary Figure S4C–F). However, introduction of the *hfqR17A* allele into the Δ*pnp* strain suppressed the defect in MgrR, McaS and ChiX levels in the Δ*pnp* background strain during late exponential phase, but not early exponential phase. For the remaining Class II sRNA, CyaR, the *hfqR17A* allele was not able to completely suppress the negative impact of the *pnp* deletion. As expected, steady state levels for all four sRNAs tested at an OD_600_ of 1.0 (Figure 4B, D, F, H) were decreased in a proximal face mutant (*hfqQ8A*) compared to WT and were not rescued in the *pnp* deletion strain (Δ*pnp hfqQ8A*); the Q8A substitution disrupts binding of both Class I and Class II sRNAs to Hfq.

**Figure 5.**
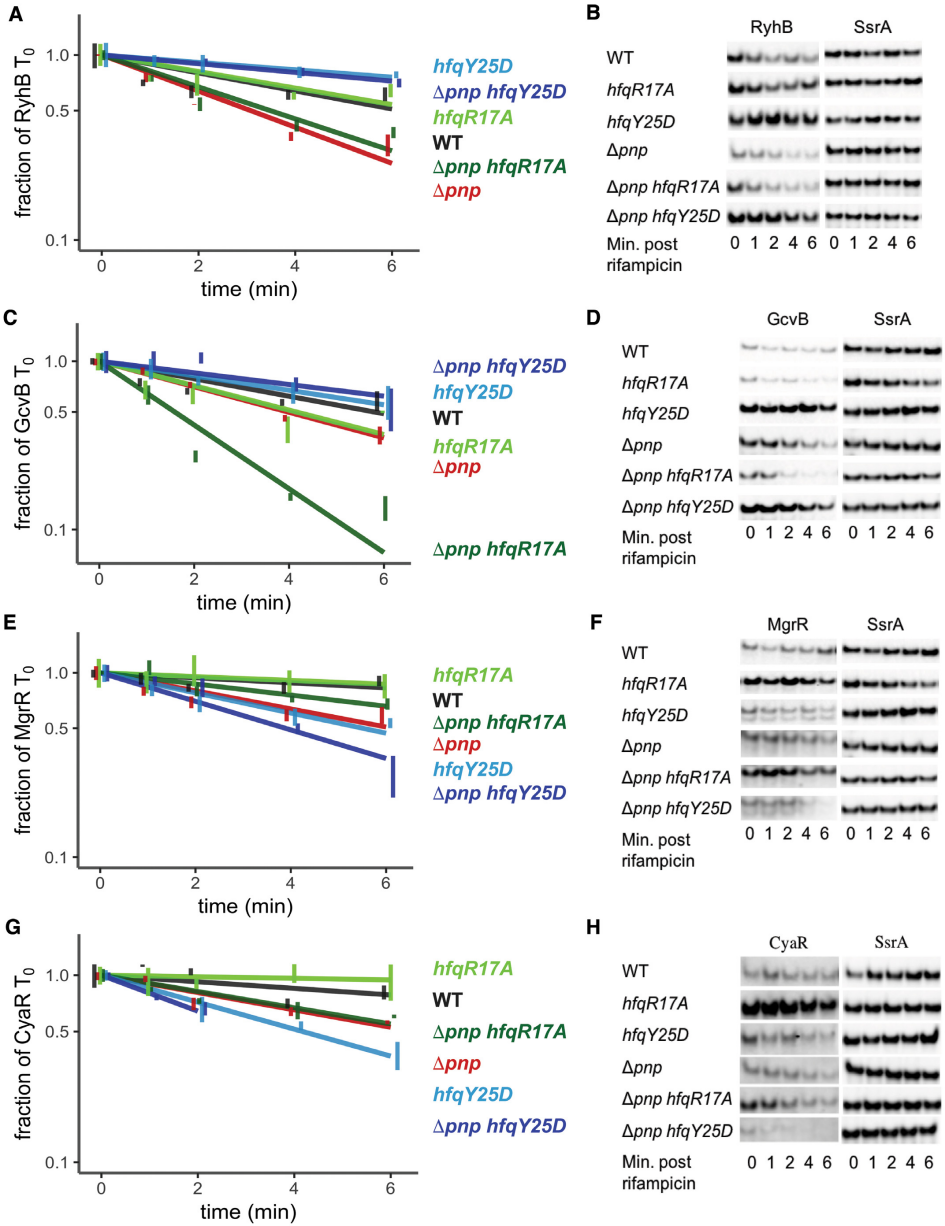
Substitutions in Hfq residues critical for binding mRNA targets suppress the stability defect of sRNAs in a Δ*pnp* strain. Overnight cultures of strain NRD1138 (WT *rph-1*) and derived strains carrying the *pnp* deletion (Δ*pnp*, DS070) and harboring mutations resulting in a substitution in a residue in the rim (R17A) or distal face (Y25D) of Hfq (*hfqR17A*, DS060; *hfqY25D*, NRD1410; Δ*pnp hfqR17A*, NRD1474; Δ*pnp hfqY25D*, NRD1478) were diluted into fresh LB medium and grown to early exponential phase (OD_600_ of 0.3). RyhB and CyaR sRNAs were induced for 15 min by the addition of 2,2′-dipyridyl or cAMP, respectively. Subsequently, rifampicin RNA stability time courses were performed as described for Figure 3. sRNA levels were examined by northern blot probing for RyhB (**A, B**), GcvB (**C, D**), MgrR (**E, F**), or CyaR (**G, H**), with representative blots shown. GcvB and MgrR levels were measured by northern blot analysis using RNA samples from the RyhB induction experiment described above. CyaR levels for the Δ*pnp hfqY25D* strain were too low at the 4 and 6 min time points to accurately measure. sRNA levels were normalized to the control RNA SsrA. The results presented in the graphs represent the mean of three independent experiments, and the error bars represent the standard error of the mean.

While the steady state levels of sRNAs are informative, these results can be difficult to interpret as expression reflects both rates of synthesis and decay, and in many cases, mRNAs encoding the transcription factors governing the synthesis of sRNAs are themselves targets of sRNA-mediated gene regulation. Thus, we next employed half-life experiments to specifically measure the impact of the *hfqY25D* and *hfqR17A* alleles and the *pnp* deletion on the turnover rates of Class I and Class II sRNAs. At an OD_600_ of 0.3, the half-lives of RyhB and GcvB increased from 3.1 and 4.1 min in the Δ*pnp* strain to 13.5 and 8.7 min in *hfqY25D* Δ*pnp* strain, respectively (Figure 5A–D, Table 1), which is comparable to their half-lives in the *hfqY25D* strain (14.2 and 7.0 min) indicating that the Y25D substitution in Hfq can overcome the negative impact on stability caused by absence of PNPase. For Class II sRNAs such as MgrR and CyaR, instability in Δ*pnp* could not be completely suppressed by introduction of *hfqR17A* (Figure 5E–H, Table 1). Due to the lack of complete suppression for these two Class II sRNAs, we also measured MgrR and CyaR half-lives at an OD_600_ of 1.0, and found similar results (Supplementary Figure S5G–J, Table 1). Finally, MgrR and CyaR stabilities were lower in strains harboring the *hfqY25D* allele, presumably due to the importance of the distal face for Class II sRNA binding (Figure 5E–H, Supplementary Figure S5G-J, Table 1). The remaining sRNAs examined (McaS, ChiX and ArcZ) were not unstable in the Δ*pnp* background strain at early or late exponential phase (Supplementary Figure S5 and data not shown), and therefore Hfq-mediated suppression could not be assessed. Thus, for some sRNAs such as RyhB and GcvB, preventing target binding to Hfq may block rapid degradation that would otherwise occur in the absence of PNPase. For other sRNAs such as CyaR, other RNA chaperones such as ProQ, which has been previously shown to bind this and other Hfq-binding sRNAs (46), may be partially redundant with Hfq; thus, preventing target binding to Hfq is not sufficient to impede the increased rate of turnover of these sRNAs in the absence of PNPase.

**Table 1.** Half-life measurements^a^

sRNA	Strain name	Avg. half-life^a^ (min) ± SE^Low^	Figure reference
CyaR OD_600_ of 0.3	Δ*pnp* + pPNPase WT	8.2 ± 1.6	Figure 3
	Δ*pnp* + pVector	5.1 ± 1.4	
	Δ*pnp*	3.4 ± 0.3	
	Δ*pnp* + pPNPase mut	3.5 ± 0.5	
RyhB OD_600_ of 0.3	Δ*pnp* + pPNPase WT	5.6 ± 0.9	Figure 3
	Δ*pnp* + pVector	3.2 ± 0.3	
	Δ*pnp*	2.3 ± 0.3	
	Δ*pnp* + pPNPase mut	2.3 ± 0.3	
RyhB OD_600_ of 0.3	WT	6.2 ± 0.9	Figure 5
	*hfqR17A*	6.5 ± 0.9	
	*hfqY25D*	14.2 ± 2.8	
	Δ*pnp*	3.1 ± 0.2	
	*hfqR17A* Δ*pnp*	3.5 ± 0.3	
	*hfqY25D* Δ*pnp*	13.5 ± 1.1	
GcvB OD_600_ of 0.3	WT	5.8 ± 0.7	Figure 5
	*hfqR17A*	4.2 ± 0.5	
	*hfqY25D*	7.0 ± 1.1	
	Δ*pnp*	4.1 ± 0.2	
	*hfqR17A* Δ*pnp*	1.6 ± 0.1	
	*hfqY25D* Δ*pnp*	8.7 ± 1.9	
MgrR OD_600_ of 0.3	WT	>20	Figure 5
	*hfqR17A*	>20	
	*hfqY25D*	5.5 ± 0.6	
	Δ*pnp*	6.1 ± 0.7	
	*hfqR17A* Δ*pnp*	9.9 ± 1.4	
	*hfqY25D* Δ*pnp*	3.9 ± 0.4	
CyaR OD_600_ of 0.3	WT	17.1 ± 4.4	Figure 5
	*hfqR17A*	>20	
	*hfqY25D*	4.2 ± 0.3	
	Δ*pnp*	6.7 ± 0.5	
	*hfqR17A* Δ*pnp*	6.9 ± 0.6	
	*hfqY25D* Δ*pnp*	3.1 ± 0.3	
ArcZ OD_600_ of 0.3	WT	>20	Supplementary Figure S5
	*hfqR17A*	>20	
	*hfqY25D*	>20	
	Δ*pnp*	17.6 ± 3.5	
	*hfqR17A* Δ*pnp*	10.7 ± 2.3	
	*hfqY25D* Δ*pnp*	>20	
McaS OD_600_ of 0.3	WT	>20	Supplementary Figure S5
	*hfqR17A*	>20	
	*hfqY25D*	10.8 ± 2.3	
	Δ*pnp*	>20	
	*hfqR17A* Δ*pnp*	>20	
	*hfqY25D* Δ*pnp*	>20	
ChiX OD_600_ of 0.3	WT	>20	Supplementary Figure S5
	*hfqR17A*	9.6 ± 2.0	
	*hfqY25D*	7.8 ± 1.2	
	Δ*pnp*	>20	
	*hfqR17A* Δ*pnp*	>20	
	*hfqY25D* Δ*pnp*	>20	
MgrR OD_600_ of 1.0	WT	17.9 ± 5.8	Supplementary Figure S5
	*hfqR17A*	>20	
	*hfqY25D*	8.9 ± 2.0	
	Δ*pnp*	14.4 ± 4.0	
	*hfqR17A* Δ*pnp*	>20	
	*hfqY25D* Δ*pnp*	7.5 ± 1.2	
CyaR OD_600_ of 1.0	WT	13.6 ± 4.0	Supplementary Figure S5
	*hfqR17A*	>20	
	*hfqY25D*	4.1 ± 0.4	
	Δ*pnp*	8.6 ± 1.9	
	*hfqR17A* Δ*pnp*	9.2 ± 1.6	
	*hfqY25D* Δ*pnp*	3.4 ± 0.4	
MgrR OD_600_ of 0.3	WT	>45	Figure 6^b^
	*mgrRmut*	>45	
	Δ*pnp*	23 ± 4.6	
	Δ*pnp mgrRmut*	27.6 ± 7.0	
RyhB OD_600_ of 0.3	WT	14.7 ± 2.5	Supplementary Figure S6
	*ryhBmut*	>20	
	*ryhBmut2*	16.4 ± 2.6	
	*ryhBdblmut*	>20	
	Δ*pnp*	3.7 ± 0.3	
	Δ*pnp ryhBmut*	2.9 ± 0.4	
	Δ*pnp ryhBmut2*	4.9 ± 1.0	
	Δ*pnp ryhBdblmut*	3.3 ± 0.4	
CyaR OD_600_ of 0.3	WT	>20	Figure 7
	*cyaRmut*	12.4 ± 3.0	
	Δ*pnp*	2.2 ± 0.3	
	Δ*pnp cyaRmut*	10.2 ± 2.1	

^a^Half-lives were determined as described in Materials and Methods. Each half-life measurement represents the average of at least three independent experiments, and ± standard error (SE) was calculated using the lower SE value.

^b^15 min time course. All other time course experiments last 6 min.

### sRNA–mRNA pairing drives sRNA decay in the absence of PNPase

To further test our hypothesis that accelerated decay of Hfq-binding sRNAs in the absence of PNPase is driven by pairing with mRNA-derived fragments, we tested how sRNA stability was affected in *pnp*^+^ and Δ*pnp* strains upon introduction of mutations within sRNAs that disrupt pairing with target mRNAs. First, we mutated two nucleotides in *ryhB* in the *pnp^+^* and Δ*pnp* strains that we previously demonstrated disrupt pairing with at least some targets of RyhB (47). We found that a RyhB mutant containing this single GC inversion (RyhBmut, Supplementary Figure S6A) within a known seed region for several targets was just as unstable as the wild-type RyhB in the Δ*pnp* strain (Supplementary Figure S6B, C). However, it is possible that the targets driving RyhB decay in the absence of PNPase do not pair with RyhB at this site, so we tested whether an additional GC inversion or the two combined GC inversions impacted RyhB stability in the Δ*pnp* strain (RyhBmut2 and RyhBdblmut, Supplementary Figure S6A). This second GC inversion is located within a motif enriched among RyhB targets (48). Both RyhB mutants were stable in the *pnp*^+^ background, but only RyhBmut2 yielded a slightly higher half-life (4.9 min) compared to RyhB (3.7 min) in the Δ*pnp* strain background (Supplementary Figure S6B–E). The inability of RyhBmut2 and RyhBdblmut to fully suppress RyhB stability in Δ*pnp* may indicate that the transcripts contributing to the accelerated decay of RyhB in Δ*pnp* bind outside these sites.

**Figure 6.**
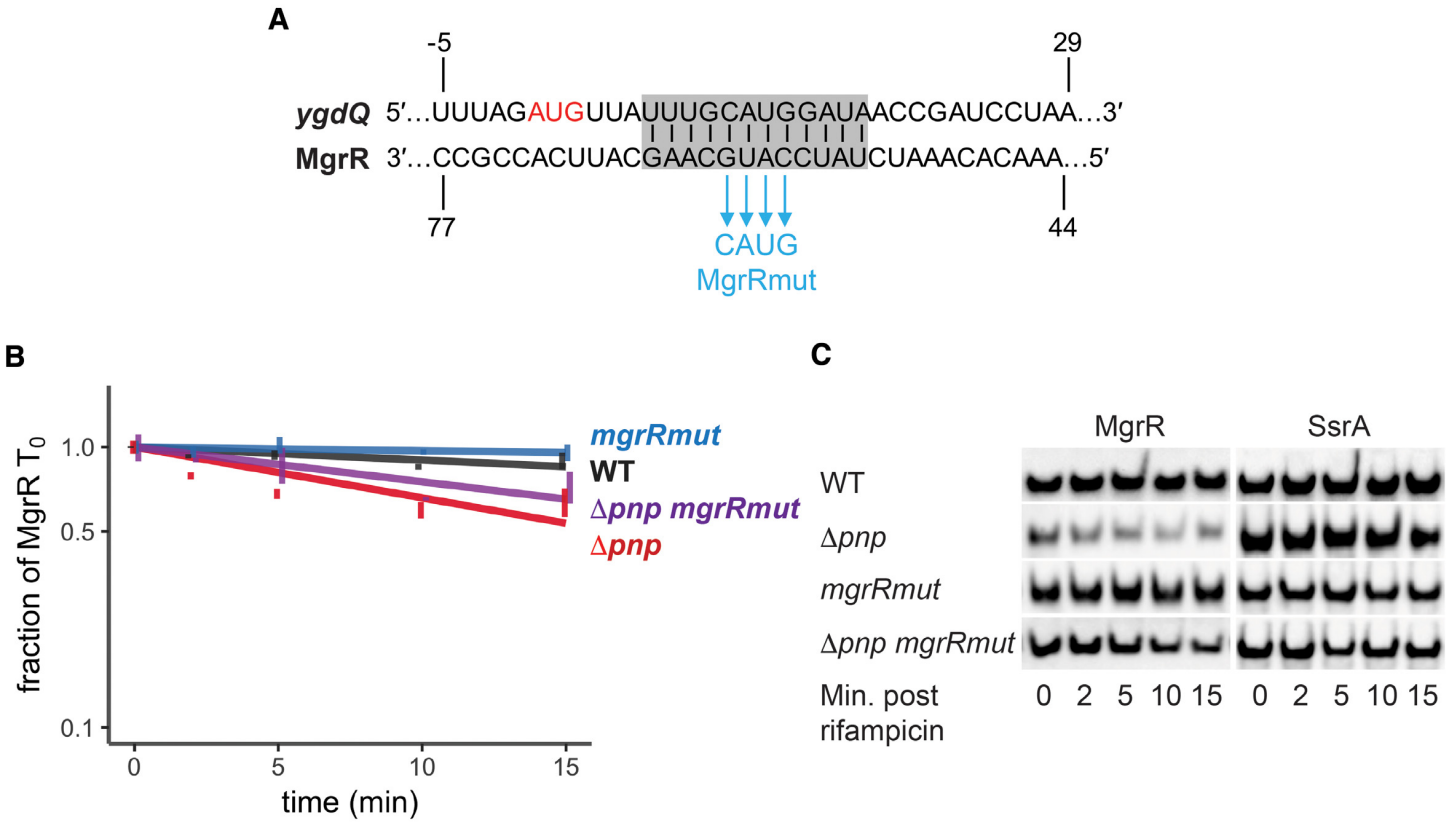
MgrR target-pairing mutations partially reduce its decay that occurs in the absence of PNPase. (**A**) Illustration showing complementarity between MgrR and *ygdQ* (highlighted in grey) and four nucleotide changes (blue) that were introduced into *mgrR* (*MgrRmut*). The start codon of *ygdQ* is depicted in red. (**B**) Stability curves of the MgrR mutant in WT and Δ*pnp* backgrounds. Overnight cultures of strain NRD1138 (WT *rph-1*) and derived strains carrying the *pnp* deletion (Δ*pnp*, NRD1139) and harboring mutations in *mgrR* (*mgrRmut*, NRD1599; Δ*pnp mgrRmut*, NRD1601) were diluted into fresh LB medium and grown to early exponential phase (OD_600_ of 0.3). Rifampicin RNA stability time courses were performed over 15 min and MgrR was examined by northern blot as described in the Materials and Methods using the probe MgrR2. MgrR levels were normalized to the control RNA SsrA and graphed as a fraction of initial MgrR level (*T*_0_). Results represent the mean of three independent experiments and the error bars represent the standard error of the mean. (**C**) Representative blots for MgrR and SsrA.

Since RyhB has greater than one hundred known targets, we therefore sought to mutate other sRNAs with fewer validated targets such as MgrR and CyaR. Four nucleotides in *mgrR* residing in the seed region of known targets (49) were replaced with the corresponding complementary bases to generate MgrRmut (Figure 6A, Table 1). The half-life was then measured over the course of 15 min following inhibition of transcription initiation. MgrR and MgrRmut levels were stable over the time course in a *pnp*^+^ strain (>45 min). MgrR showed a decrease in stability in the *pnp* deletion strain (half-life of 23 min); however, the four nucleotide substitution in the seed region of MgrR led to a modest increase in the MgrR half-life (27.6 min) in the *pnp* deletion background (Δ*pnp mgrRmut*), which may indicate a partial suppression of the *pnp* deletion phenotype.

**Figure 7.**
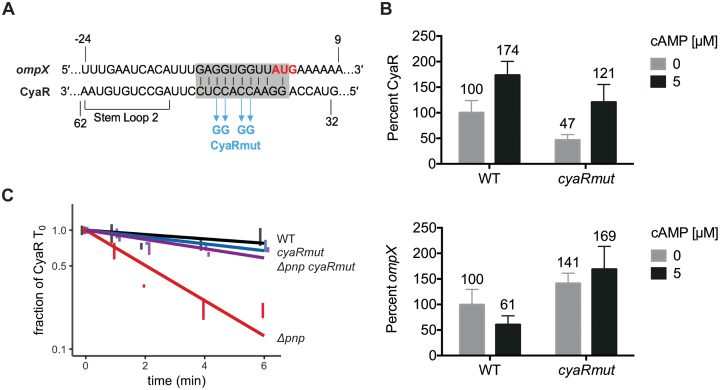
CyaR target-pairing drives its decay in the absence of PNPase. (**A**) The indicated point mutations were introduced into CyaR to generate CyaR mut. The CyaR-*ompX* pairing region is highlighted by the grey box. (**B**) Comparison of the levels of CyaR (top) and *ompX* (bottom) after induction of CyaR in a strain carrying CyaR wild-type (WT *rph-1*, NRD1138) or a derived strain carrying the CyaR pairing mutation (*cyaRmut*, TC468). Total RNA was collected from cultures 15 min after addition of 0 or 5 mM cyclic AMP (cAMP) and assessed by northern blot. Error bars indicate mean and standard error of three replicates. (**C**) Stability curves of the CyaR wild-type with native *pnp* (WT *rph-1*, NRD1138) and derived strains carrying Δ*pnp* (NRD1139) or the CyaR pairing mutant with native *pnp* (TC468) or Δ*pnp* (TC476). Expression of CyaR was induced by addition of cAMP for 15 min, followed by addition of rifampicin and collection of total RNA at the indicated time points as described for Figure 3. Lines indicate best-fit exponential decay curves of three replicates, and error bars indicate the mean and standard error of each time point. Representative northern blots for B and C are shown in Supplementary Figure S7.

Next we examined whether sRNA–mRNA target pairing accelerates CyaR turnover in the absence of PNPase. Target-pairing mutations were introduced into the native *cyaR* genomic locus by replacing four nucleotides within the first hairpin loop of CyaR previously identified as a pairing region for several CyaR targets (50) with the corresponding complementary bases (Figure 7A). We compared the expression of CyaRmut to wild-type CyaR in *a pnp^+^* strain in the presence or absence of exogenous addition of its inducer cAMP, which binds to CRP and consequently promotes transcription of this sRNA. CyaRmut expression was 47% and 70% of wild-type CyaR levels under non-inducing and inducing conditions, respectively (Supplementary Figure S7A, Figure 7B). In contrast with the wild-type CyaR, expression of the CyaR mutant did not lead to decreased levels of *ompX* (Figure 7B, Supplementary Figure S7A) indicating that the mutation was successful in disrupting pairing with a known CyaR target mRNA. The stability of the CyaR pairing mutant was next compared to the wild-type CyaR in the presence and absence of PNPase. Remarkably, disruption of target pairing nearly completely suppressed CyaR instability in the absence of PNPase (Figure 7C). This result is consistent with a model in which PNPase stabilizes sRNAs and promotes sRNA-mediated gene regulation by helping to degrade RNAs that would otherwise lead to non-productive base-pairing with sRNAs.

## DISCUSSION

We previously reported that the highly conserved 3′-to-5′ exoribonuclease PNPase can paradoxically act to stabilize and promote the function of many Hfq-binding sRNAs (29,31,32). Moreover, we (31,32) and others (30) have shown that PNPase does not degrade Hfq-bound sRNAs but can degrade the unbound sRNAs. Additionally, PNPase forms a ternary construct with Hfq *in vitro* that can be mediated by sRNAs (51). However, the precise mechanism by which PNPase promotes sRNA stability has remained unresolved. Here, we used a combination of high throughput RNA-sequencing, co-immunoprecipitation experiments with Hfq and PNPase, and RNA half-life experiments with Hfq and sRNA mutants to further refine our understanding of the substrate specificity of this exoribonuclease and the mechanism by which it stabilizes some Hfq-bound sRNAs.

### PNPase is required for the degradation of specific mRNA-derived fragments

Although PNPase was discovered more than six decades ago, there has been a paucity of work published on the substrate specificity of this enzyme. In vitro studies primarily using artificial RNAs or fragments of natural substrates demonstrated that PNPase is a processive 3′-to-5′ exoribonuclease, which can rapidly degrade through single-stranded RNA sequences or double-stranded RNA sequences as long as a single-stranded sequence of 7–9 nt is present at the 3′ end to facilitate initial loading of this RNase onto its substrate (52,53). Once bound to an RNA substrate, PNPase can degrade through double-stranded sequences at a rate of 121 nt/s (54). Additional *in vitro* studies using artificial RNA substrates revealed that PNPase is inhibited by very stable GC-rich stem-loop structures. *In vivo* studies have focused on the activity of PNPase on particular substrates such as rRNAs (55,56) or tRNAs (57–59), but the knowledge gained from these studies is more informative about the steps in the processing of these transcripts rather than the specificity of this enzyme.

We sought to garner more details about the specificity of this enzyme by comparing the transcript profiles of a wild-type and Δ*pnp* strain via short RNA-seq and mRNA-seq. Using a short RNA-seq based approach, we found 106 distinct RNA fragments that significantly accumulated in the absence of PNPase (Figure 1C) suggesting that PNPase is the only one of the seven exoribonucleases (RNase T, RNase R, RNase II, RNase D, RNase PH, oligoribonuclease and PNPase) in *E. coli* that can efficiently degrade these RNA snippets. Examination of six transcripts (*yqaE, dsbB, chiP, miaA, hemN* and *cfa*) that were identified by short RNA-seq as up-regulated in the Δ*pnp* strain compared to the wild-type strain revealed a significant increase in the corresponding RNA fragments in the Δ*pnp* strain by northern blot analysis (Figure 1) but very little difference in the levels of the full-length mRNA (Supplementary Figure S1). Consistent with the idea that PNPase uniquely contributes to the decay of particular mRNA-derived fragments without impacting the levels of full-length mRNAs, just one of the 106 fragments was associated with any gene detected in the mRNA-seq analysis as significantly differentially expressed in the Δ*pnp* strain as compared to its *pnp^+^* parent strain.

The mRNA-derived fragments significantly up-regulated in the Δ*pnp* strain relative to the wild-type strain could be substrates that are only efficiently degraded by PNPase; alternatively, the levels of these RNAs may be indirectly affected by loss of PNPase. To differentiate between these possibilities, we assessed whether the active site residues of PNPase were required for decay of these mRNA-derived fragments and tested the ability of PNPase to bind these RNAs. Interestingly, all five RNA fragments that we tested (*yqaE* 5′ UTR, *miaA* CDS, *hemN* 5′ UTR, *dsbB* CDS and *cfa* 5′ UTR) accumulated in an *E. coli* strain expressing the active site mutant form of PNPase (Figure 3A) and co-immunoprecipitated with this enzymatically inactive mutant protein (Figure 3B) consistent with these RNA fragments being substrates degraded by PNPase. An interesting feature found in many of these RNA fragments is that they are highly structured (Supplementary Figure S8).

### Molecular mechanism of PNPase-mediated sRNA stabilization

**Figure 8.**
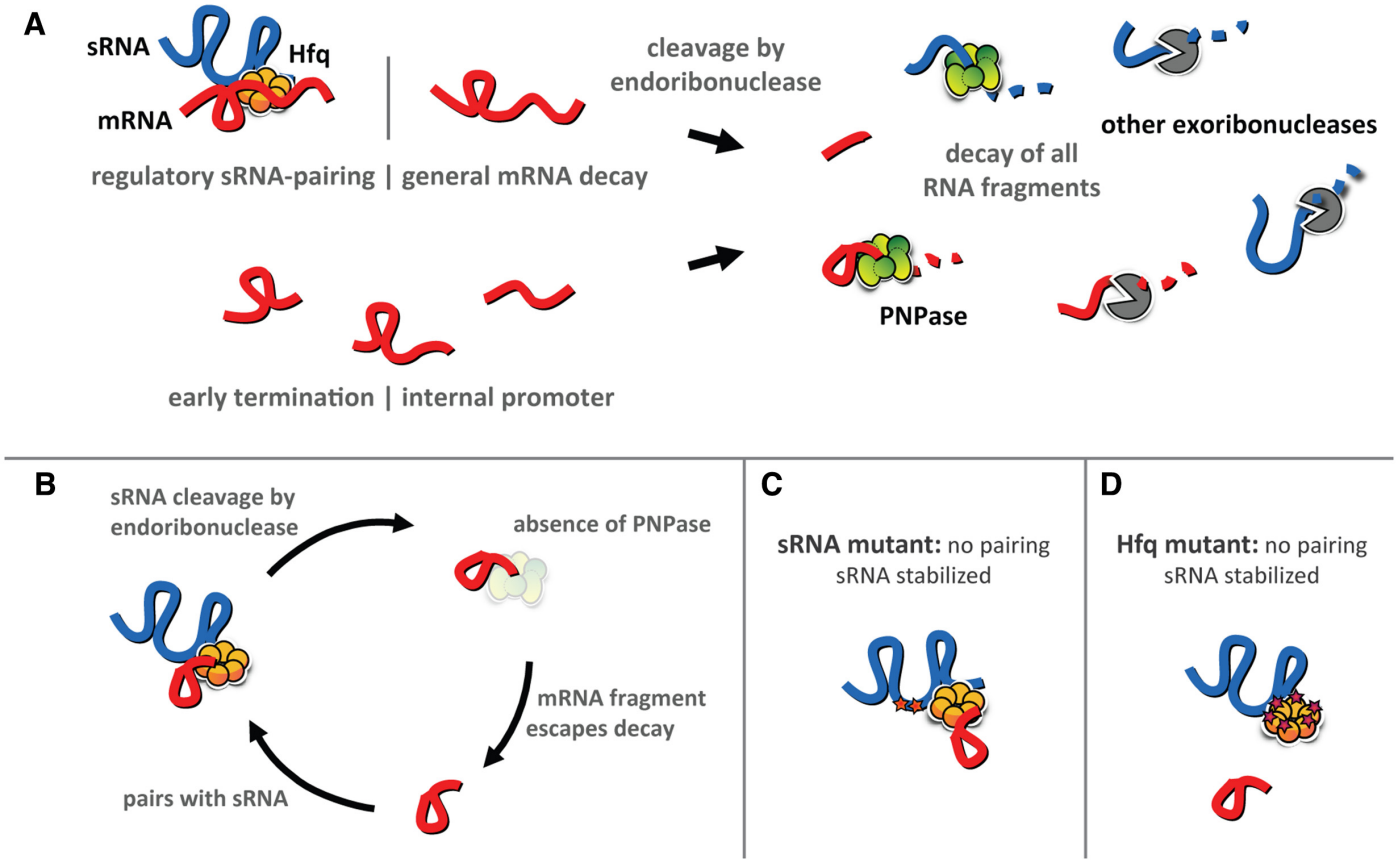
A model for the role of PNPase in sRNA-mediated gene regulation. (**A**) mRNA fragments are first produced by endoribonuclease cleavage due to regulatory sRNA pairing or general mRNA decay, or directly via early transcription termination or by internal promoters. In the presence of PNPase, mRNA fragments are completely digested. (**B**) In the absence of PNPase, some mRNA fragments escape decay by other exoribonucleases. Those that retain Hfq binding and sRNA pairing sequences may interact with sRNAs and promote their cleavage by endoribonucleases. (C, D) In the absence of PNPase, sRNA stability may be restored by disrupting sRNA–mRNA pairing, either by mutating the pairing region of the sRNA (**C**) or by mutating the corresponding mRNA-binding site of Hfq (**D**).

Another common feature of many but not all of these fragments that are dependent on PNPase for decay is that that these RNAs are able to bind to Hfq (Figure 2) and are derived from mRNAs that are targets of Hfq-dependent sRNAs (Figure 1C). These results along with our findings reported previously (29,31,32) and here (Figure 5) that PNPase inhibits the decay of Hfq-dependent sRNAs indicated to us that PNPase may be stabilizing some sRNAs by degrading mRNA fragments that could pair with and drive the decay of their cognate sRNAs in an Hfq-dependent manner. In this model, the mRNA fragments would remain intact to act on additional sRNA molecules (Figure 8). This mechanism would be analogous to how the 3′ external transcribed spacer of the *glyW*-*cysT-leuZ* tRNA precursor has been reported to act on the Hfq-dependent sRNA RyhB (47,60).

We predicted that if this was the mechanism by which PNPase mediated sRNA stabilization, then substitutions in Hfq of key residues in the distal face and rim would suppress the stability defect in the Δ*pnp* strain of Class I sRNAs such as RyhB and GcvB and Class II sRNAs such as MgrR and CyaR, respectively, by preventing pairing to mRNA-derived fragments. As mentioned above, Hfq has three main binding surfaces: the proximal face, distal face, and rim. Class I sRNAs bind the proximal face and rim, and Class II sRNAs bind the distal face; the cognate mRNA targets of Class I and Class II sRNAs bind the distal face and the rim, respectively (21,23). Consistent with this prediction, we found that introduction of the *hfqY25D* allele that produces an Hfq distal face mutant (Y25D) into the Δ*pnp* strain completely suppressed the stability defect of RyhB and GcvB (Figure 5A–D). Moreover, introduction of the *hfqR17A* allele encoding the Hfq rim mutant (R17A) into the Δ*pnp* strain reduced MgrR turnover, albeit not to wild-type levels (Figure 5E, F). Interestingly, CyaR stability could not be rescued in Δ*pnp hfqR17A* (Figure 5G, H) even though introduction of mutations in *cyaR* that disrupted pairing with its targets (Figure 7A, B) into the Δ*pnp* strain did restore CyaR stability to levels observed in a *pnp*^+^ strain (Figure 7C). This inability of the *hfqR17A* allele to rescue the CyaR stability defect caused by the *pnp* deletion could possibly be due to a redundant role for the RNA chaperone ProQ in facilitating CyaR annealing to mRNA-derived fragments; consistent with this speculative model, ProQ was previously shown to bind CyaR and some of its target mRNAs in *Salmonella enterica* serovar Typhimurium (46).

The mutations that we introduced into *ryhB* to disrupt pairing of RyhB with its target mRNAs did not restore its stability in the Δ*pnp* strain to WT levels (Supplementary Figure S6). One possible explanation for this result is that RyhB has additional regions that can pair with other target mRNAs (48), and more extensive mutagenesis of RyhB would be necessary to disrupt its interactions with all of its >100 distinct targets (1,41,60,61). Another possibility is that PNPase has different mechanisms of stabilizing distinct sRNAs. For example, PNPase may stabilize CyaR by degrading mRNA-derived fragments that drive its decay, but could block RyhB decay through another mechanism possibly by forming a protective ribonucleoprotein complex with this RNA and Hfq as we proposed previously (31).

What are the particular mRNA-derived fragments that could be driving the decay of CyaR and other Hfq-binding sRNAs in the absence of PNPase? In our short RNA-seq data set (Supplementary Table S4), we find in the Δ*pnp* strain up-regulation of several different targets for each Hfq-binding sRNA that we have examined. Thus, we predict that there may be several distinct mRNA fragments driving the decay of Hfq and PNPase-dependent sRNAs in the absence of PNPase. Additionally, different target mRNAs may be expressed under different conditions, which would in turn affect which specific fragments are present and could impact sRNA stability. For instance, in our experiments testing steady state levels of sRNAs in the Δ*pnp* strain, introduction of the *hfqY25D* or *hfqR17A* allele yielded larger increases in steady state levels in late exponential phase cultures compared to early exponential phase (Figure 4, Supplementary Figure S4). Likewise, Dressaire *et al.* (62) found for late stationary phase cultures that the absence of PNPase resulted in global mRNA destabilization, and indeed, in stationary phase cultures (OD_600_ of 2.0) some sRNAs such as CyaR or MicA are not destabilized by the loss of PNPase (30,32); MicA is actually degraded by PNPase during stationary phase (30,32,43,63). Thus, it is possible that the majority of transcripts driving the decay of particular sRNAs in the absence of PNPase are more abundant during late exponential phase compared to early exponential phase or late stationary phase. Future studies examining the generation of these mRNA fragments under different growth conditions would help to clarify if they have a role in these differences.

Finally, it has been proposed that upon sRNA–mRNA annealing the C-terminal domain of Hfq acts to displace the sRNA–mRNAs from this chaperone (64). This postulate is based on in vitro data demonstrating that a C-terminally truncated form of Hfq has reduced displacement of model substrates upon RNA–RNA annealing as compared to full-length Hfq (64). While the C-terminal domain may participate in sRNA–mRNA displacement from Hfq, we speculate that PNPase may be responsible for removing and degrading Hfq-bound mRNA fragments following endonucleolytic cleavage by RNase E. This would be akin to the function that PNPase plays with regards to the Y-RNA, Ro auto-antigen and rRNAs in *Deinococcus radiodurans*, where the Y-RNA acts as the tether between PNPase and Ro antigen, and PNPase degrades rRNAs fed to it by Ro (65,66). Indeed, we previously demonstrated that Hfq-binding sRNAs act as a tether between Hfq and PNPase in vitro, and that PNPase is unable to degrade Hfq-bound sRNAs (31). In this model, upon sRNA–mRNA pairing, RNase E would cleave the mRNA and the sRNA and mRNA-derived fragment would be displaced from Hfq as PNPase degrades this piece of RNA. In future work, we plan on testing this model.

## DATA AVAILABILITY

Gene expression data have been deposited with NCBI Gene Expression Omnibus (GEO) under accession number GSE125368 and will be released upon publication of this manuscript.

## Supplementary Material

gkz616_Supplemental_Files

## References

[1] Masse E., Vanderpool C.K., Gottesman S. Effect of RyhB small RNA on global iron use in *Escherichia coli*. J. Bacteriol. 2005; 187:6962–6971. 16199566 10.1128/JB.187.20.6962-6971.2005 PMC1251601

[2] Gottesman S., Storz G. Bacterial small RNA regulators: versatile roles and rapidly evolving variations. Cold Spring Harb. Perspect. Biol. 2011; 3:a003798. 20980440 10.1101/cshperspect.a003798 PMC3225950

[3] Jorgensen M.G., Thomason M.K., Havelund J., Valentin-Hansen P., Storz G. Dual function of the McaS small RNA in controlling biofilm formation. Genes Dev. 2013; 27:1132–1145. 23666921 10.1101/gad.214734.113 PMC3672647

[4] Parker A., Cureoglu S., De Lay N., Majdalani N., Gottesman S. Alternative pathways for *Escherichia coli* biofilm formation revealed by sRNA overproduction. Mol. Microbiol. 2017; 105:309–325. 28470798 10.1111/mmi.13702 PMC5510166

[5] Vanderpool C.K., Gottesman S. Involvement of a novel transcriptional activator and small RNA in post-transcriptional regulation of the glucose phosphoenolpyruvate phosphotransferase system. Mol. Microbiol. 2004; 54:1076–1089. 15522088 10.1111/j.1365-2958.2004.04348.x

[6] Masse E., Salvail H., Desnoyers G., Arguin M. Small RNAs controlling iron metabolism. Curr. Opin. Microbiol. 2007; 10:140–145. 17383226 10.1016/j.mib.2007.03.013

[7] Thomason M.K., Fontaine F., De Lay N., Storz G. A small RNA that regulates motility and biofilm formation in response to changes in nutrient availability in *Escherichia coli*. Mol. Microbiol. 2012; 84:17–35. 22289118 10.1111/j.1365-2958.2012.07965.x PMC3312966

[8] De Lay N., Gottesman S. A complex network of small non-coding RNAs regulate motility in *Escherichia coli*. Mol. Microbiol. 2012; 86:524–538. 22925049 10.1111/j.1365-2958.2012.08209.x PMC7458410

[9] Hebrard M., Kroger C., Srikumar S., Colgan A., Handler K., Hinton J.C. sRNAs and the virulence of *Salmonella enterica* serovar Typhimurium. RNA Biol. 2012; 9:437–445. 22546935 10.4161/rna.20480 PMC3384567

[10] Zhang S., Liu S., Wu N., Yuan Y., Zhang W., Zhang Y. Small Non-coding RNA RyhB mediates persistence to multiple antibiotics and stresses in uropathogenic *Escherichia coli* by reducing cellular metabolism. Front. Microbiol. 2018; 9:136. 29467745 10.3389/fmicb.2018.00136 PMC5808207

[11] Moller T., Franch T., Hojrup P., Keene D.R., Bachinger H.P., Brennan R.G., Valentin-Hansen P. Hfq: a bacterial Sm-like protein that mediates RNA-RNA interaction. Mol. Cell. 2002; 9:23–30. 11804583 10.1016/s1097-2765(01)00436-1

[12] Zhang A., Wassarman K.M., Rosenow C., Tjaden B.C., Storz G., Gottesman S. Global analysis of small RNA and mRNA targets of Hfq. Mol. Microbiol. 2003; 50:1111–1124. 14622403 10.1046/j.1365-2958.2003.03734.x

[13] Vogel J., Luisi B.F. Hfq and its constellation of RNA. Nat. Rev. Microbiol. 2011; 9:578–589. 21760622 10.1038/nrmicro2615 PMC4615618

[14] Chao Y., Papenfort K., Reinhardt R., Sharma C.M., Vogel J. An atlas of Hfq-bound transcripts reveals 3′ UTRs as a genomic reservoir of regulatory small RNAs. EMBO J. 2012; 31:4005–4019. 22922465 10.1038/emboj.2012.229 PMC3474919

[15] Panja S., Woodson S.A. Hfq proximity and orientation controls RNA annealing. Nucleic Acids Res. 2012; 40:8690–8697. 22761405 10.1093/nar/gks618 PMC3458560

[16] Schumacher M.A., Pearson R.F., Moller T., Valentin-Hansen P., Brennan R.G. Structures of the pleiotropic translational regulator Hfq and an Hfq-RNA complex: a bacterial Sm-like protein. EMBO J. 2002; 21:3546–3556. 12093755 10.1093/emboj/cdf322 PMC126077

[17] Mikulecky P.J., Kaw M.K., Brescia C.C., Takach J.C., Sledjeski D.D., Feig A.L. *Escherichia coli* Hfq has distinct interaction surfaces for DsrA, rpoS and poly(A) RNAs. Nat. Struct. Mol. Biol. 2004; 11:1206–1214. 15531892 10.1038/nsmb858 PMC3071270

[18] Link T.M., Valentin-Hansen P., Brennan R.G. Structure of *Escherichia coli* Hfq bound to polyriboadenylate RNA. Proc. Natl. Acad. Sci. U.S.A. 2009; 106:19292–19297. 19889981 10.1073/pnas.0908744106 PMC2773200

[19] Robinson K.E., Orans J., Kovach A.R., Link T.M., Brennan R.G. Mapping Hfq-RNA interaction surfaces using tryptophan fluorescence quenching. Nucleic Acids Res. 2014; 42:2736–2749. 24288369 10.1093/nar/gkt1171 PMC3936774

[20] Sauer E., Schmidt S., Weichenrieder O. Small RNA binding to the lateral surface of Hfq hexamers and structural rearrangements upon mRNA target recognition. Proc. Natl. Acad. Sci. U.S.A. 2012; 109:9396–9401. 22645344 10.1073/pnas.1202521109 PMC3386104

[21] Zhang A., Schu D.J., Tjaden B.C., Storz G., Gottesman S. Mutations in interaction surfaces differentially impact *E. coli* Hfq association with small RNAs and their mRNA targets. J. Mol. Biol. 2013; 425:3678–3697. 23318956 10.1016/j.jmb.2013.01.006 PMC3640674

[22] Dimastrogiovanni D., Frohlich K.S., Bandyra K.J., Bruce H.A., Hohensee S., Vogel J., Luisi B.F. Recognition of the small regulatory RNA RydC by the bacterial Hfq protein. Elife. 2014; 3:doi:10.7554/eLife.05375. 10.7554/eLife.05375 PMC4337610 25551292

[23] Schu D.J., Zhang A., Gottesman S., Storz G. Alternative Hfq-sRNA interaction modes dictate alternative mRNA recognition. EMBO J. 2015; 34:2557–2573. 26373314 10.15252/embj.201591569 PMC4609186

[24] Lease R.A., Cusick M.E., Belfort M. Riboregulation in *Escherichia coli*: DsrA RNA acts by RNA:RNA interactions at multiple loci. Proc. Natl. Acad. Sci. U.S.A. 1998; 95:12456–12461. 9770507 10.1073/pnas.95.21.12456 PMC22852

[25] Bouvier M., Sharma C.M., Mika F., Nierhaus K.H., Vogel J. Small RNA binding to 5′ mRNA coding region inhibits translational initiation. Mol. Cell. 2008; 32:827–837. 19111662 10.1016/j.molcel.2008.10.027

[26] Masse E., Escorcia F.E., Gottesman S. Coupled degradation of a small regulatory RNA and its mRNA targets in *Escherichia coli*. Genes Dev. 2003; 17:2374–2383. 12975324 10.1101/gad.1127103 PMC218075

[27] Pfeiffer V., Papenfort K., Lucchini S., Hinton J.C., Vogel J. Coding sequence targeting by MicC RNA reveals bacterial mRNA silencing downstream of translational initiation. Nat. Struct. Mol. Biol. 2009; 16:840–846. 19620966 10.1038/nsmb.1631

[28] Bandyra K.J., Said N., Pfeiffer V., Gorna M.W., Vogel J., Luisi B.F. The seed region of a small RNA drives the controlled destruction of the target mRNA by the endoribonuclease RNase E. Mol. Cell. 2012; 47:943–953. 22902561 10.1016/j.molcel.2012.07.015 PMC3469820

[29] De Lay N., Gottesman S. Role of polynucleotide phosphorylase in sRNA function in *Escherichia coli*. RNA. 2011; 17:1172–1189. 21527671 10.1261/rna.2531211 PMC3096048

[30] Andrade J.M., Pobre V., Matos A.M., Arraiano C.M. The crucial role of PNPase in the degradation of small RNAs that are not associated with Hfq. RNA. 2012; 18:844–855. 22355164 10.1261/rna.029413.111 PMC3312570

[31] Bandyra K.J., Sinha D., Syrjanen J., Luisi B.F., De Lay N.R. The ribonuclease polynucleotide phosphorylase can interact with small regulatory RNAs in both protective and degradative modes. RNA. 2016; 22:360–372. 26759452 10.1261/rna.052886.115 PMC4748814

[32] Cameron T.A., De Lay N.R. The phosphorolytic exoribonucleases polynucleotide phosphorylase and RNase PH stabilize sRNAs and facilitate regulation of their mRNA Targets. J. Bacteriol. 2016; 198:3309–3317. 27698082 10.1128/JB.00624-16 PMC5116934

[33] Cameron T.A., Matz L.M., De Lay N.R. Polynucleotide phosphorylase: not merely an RNase but a pivotal post-transcriptional regulator. PLos Genet. 2018; 14:e1007654. 30307990 10.1371/journal.pgen.1007654 PMC6181284

[34] Martin M. Cutadapt removes adapter sequences from high-throughput sequencing reads. EMBnet J. 2011; 17:3.

[35] Li H. Aligning sequence reads, clone sequences and assembly contigs with BWA-MEM. 2013; arXiv doi: https://arxiv.org/abs/1303.3997, 16 March 2013, preprint: not peer reviewed.

[36] Li H., Handsaker B., Wysoker A., Fennell T., Ruan J., Homer N., Marth G., Abecasis G., Durbin R. Genome Project Data Processing, S The sequence Alignment/Map format and SAMtools. Bioinformatics. 2009; 25:2078–2079. 19505943 10.1093/bioinformatics/btp352 PMC2723002

[37] Liao Y., Smyth G.K., Shi W. featureCounts: an efficient general purpose program for assigning sequence reads to genomic features. Bioinformatics. 2014; 30:923–930. 24227677 10.1093/bioinformatics/btt656

[38] Love M.I., Huber W., Anders S. Moderated estimation of fold change and dispersion for RNA-seq data with DESeq2. Genome Biol. 2014; 15:550. 25516281 10.1186/s13059-014-0550-8 PMC4302049

[39] Thomason M.K., Bischler T., Eisenbart S.K., Forstner K.U., Zhang A., Herbig A., Nieselt K., Sharma C.M., Storz G. Global transcriptional start site mapping using differential RNA sequencing reveals novel antisense RNAs in *Escherichia coli*. J. Bacteriol. 2015; 197:18–28. 25266388 10.1128/JB.02096-14 PMC4288677

[40] Keseler I.M., Mackie A., Santos-Zavaleta A., Billington R., Bonavides-Martinez C., Caspi R., Fulcher C., Gama-Castro S., Kothari A., Krummenacker M. et al. The EcoCyc database: reflecting new knowledge about *Escherichia coli* K-12. Nucleic Acids Res. 2017; 45:D543–D550. 27899573 10.1093/nar/gkw1003 PMC5210515

[41] Melamed S., Peer A., Faigenbaum-Romm R., Gatt Y.E., Reiss N., Bar A., Altuvia Y., Argaman L., Margalit H. Global mapping of small RNA-Target interactions in bacteria. Mol. Cell. 2016; 63:884–897. 27588604 10.1016/j.molcel.2016.07.026 PMC5145812

[42] Pobre V., Arraiano C.M. Next generation sequencing analysis reveals that the ribonucleases RNase II, RNase R and PNPase affect bacterial motility and biofilm formation in *E. coli*. BMC Genomics. 2015; 16:72. 25757888 10.1186/s12864-015-1237-6 PMC4335698

[43] Viegas S.C., Pfeiffer V., Sittka A., Silva I.J., Vogel J., Arraiano C.M. Characterization of the role of ribonucleases in *Salmonella* small RNA decay. Nucleic Acids Res. 2007; 35:7651–7664. 17982174 10.1093/nar/gkm916 PMC2190706

[44] Soper T.J., Woodson S.A. The rpoS mRNA leader recruits Hfq to facilitate annealing with DsrA sRNA. RNA. 2008; 14:1907–1917. 18658123 10.1261/rna.1110608 PMC2525945

[45] Lease R.A., Woodson S.A. Cycling of the Sm-like protein Hfq on the DsrA small regulatory RNA. J. Mol. Biol. 2004; 344:1211–1223. 15561140 10.1016/j.jmb.2004.10.006

[46] Holmqvist E., Li L., Bischler T., Barquist L., Vogel J. Global maps of ProQ binding in vivo reveal target recognition via RNA structure and stability control at mRNA 3′ ends. Mol. Cell. 2018; 70:971–982. 29804828 10.1016/j.molcel.2018.04.017

[47] Sinha D., Matz L.M., Cameron T.A., De Lay N.R. Poly(A) polymerase is required for RyhB sRNA stability and function in *Escherichia coli*. RNA. 2018; 24:1496–1511. 30061117 10.1261/rna.067181.118 PMC6191717

[48] Waters S.A., McAteer S.P., Kudla G., Pang I., Deshpande N.P., Amos T.G., Leong K.W., Wilkins M.R., Strugnell R., Gally D.L. et al. Small RNA interactome of pathogenic *E. coli* revealed through crosslinking of RNase E. EMBO J. 2017; 36:374–387. 27836995 10.15252/embj.201694639 PMC5286369

[49] Moon K., Gottesman S. A PhoQ/P-regulated small RNA regulates sensitivity of *Escherichia coli* to antimicrobial peptides. Mol. Microbiol. 2009; 74:1314–1330. 19889087 10.1111/j.1365-2958.2009.06944.x PMC2841474

[50] De Lay N., Gottesman S. The Crp-activated small noncoding regulatory RNA CyaR (RyeE) links nutritional status to group behavior. J. Bacteriol. 2009; 191:461–476. 18978044 10.1128/JB.01157-08 PMC2620814

[51] Bandyra K.J., Sinha D., Syrjanen J., Luisi B.F., De Lay N.R. The ribonuclease polynucleotide phosphorylase can interact with small regulatory RNAs in both protective and degradative modes. RNA. 2016; 22:360–372. 26759452 10.1261/rna.052886.115 PMC4748814

[52] Stickney L.M., Hankins J.S., Miao X., Mackie G.A. Function of the conserved S1 and KH domains in polynucleotide phosphorylase. J. Bacteriol. 2005; 187:7214–7221. 16237005 10.1128/JB.187.21.7214-7221.2005 PMC1272994

[53] Fernandez-Ramirez F., Bermudez-Cruz R.M., Montanez C. Nucleic acid and protein factors involved in Escherichia coli polynucleotide phosphorylase function on RNA. Biochimie. 2010; 92:445–454. 20114069 10.1016/j.biochi.2010.01.004

[54] Fazal F.M., Koslover D.J., Luisi B.F., Block S.M. Direct observation of processive exoribonuclease motion using optical tweezers. Proc. Natl. Acad. Sci. U.S.A. 2015; 112:15101–15106. 26598710 10.1073/pnas.1514028112 PMC4679025

[55] Cheng Z.F., Deutscher M.P. Quality control of ribosomal RNA mediated by polynucleotide phosphorylase and RNase R. Proc. Natl. Acad. Sci. U.S.A. 2003; 100:6388–6393. 12743360 10.1073/pnas.1231041100 PMC164456

[56] Sulthana S., Basturea G.N., Deutscher M.P. Elucidation of pathways of ribosomal RNA degradation: an essential role for RNase E. RNA. 2016; 22:1163–1171. 27298395 10.1261/rna.056275.116 PMC4931109

[57] Li Z., Deutscher M.P. The role of individual exoribonucleases in processing at the 3′ end of *Escherichia coli* tRNA precursors. J. Biol. Chem. 1994; 269:6064–6071. 7509797

[58] Mohanty B.K., Kushner S.R. Processing of the *Escherichia coli leuX* tRNA transcript, encoding tRNA(Leu5), requires either the 3′→5′ exoribonuclease polynucleotide phosphorylase or RNase P to remove the Rho-independent transcription terminator. Nucleic Acids Res. 2010; 38:597–607. 19906695 10.1093/nar/gkp997 PMC2811032

[59] Mohanty B.K., Petree J.R., Kushner S.R. Endonucleolytic cleavages by RNase E generate the mature 3′ termini of the three proline tRNAs in *Escherichia coli*. Nucleic Acids Res. 2016; 44:6350–6362. 27288443 10.1093/nar/gkw517 PMC5291269

[60] Lalaouna D., Carrier M.C., Semsey S., Brouard J.S., Wang J., Wade J.T., Masse E. A 3′ external transcribed spacer in a tRNA transcript acts as a sponge for small RNAs to prevent transcriptional noise. Mol. Cell. 2015; 58:393–405. 25891076 10.1016/j.molcel.2015.03.013

[61] Wang J., Rennie W., Liu C., Carmack C.S., Prevost K., Caron M.P., Masse E., Ding Y., Wade J.T. Identification of bacterial sRNA regulatory targets using ribosome profiling. Nucleic Acids Res. 2015; 43:10308–10320. 26546513 10.1093/nar/gkv1158 PMC4666370

[62] Dressaire C., Pobre V., Laguerre S., Girbal L., Arraiano C.M., Cocaign-Bousquet M. PNPase is involved in the coordination of mRNA degradation and expression in stationary phase cells of *Escherichia coli*. BMC Genomics. 2018; 19:848. 30486791 10.1186/s12864-018-5259-8 PMC6264599

[63] Andrade J.M., Arraiano C.M. PNPase is a key player in the regulation of small RNAs that control the expression of outer membrane proteins. RNA. 2008; 14:543–551. 18203924 10.1261/rna.683308 PMC2248267

[64] Santiago-Frangos A., Kavita K., Schu D.J., Gottesman S., Woodson S.A. C-terminal domain of the RNA chaperone Hfq drives sRNA competition and release of target RNA. Proc. Natl. Acad. Sci. U.S.A. 2016; 113:E6089–E6096. 27681631 10.1073/pnas.1613053113 PMC5068269

[65] Chen X., Taylor D.W., Fowler C.C., Galan J.E., Wang H.W., Wolin S.L. An RNA degradation machine sculpted by Ro autoantigen and noncoding RNA. Cell. 2013; 153:166–177. 23540697 10.1016/j.cell.2013.02.037 PMC3646564

[66] Wurtmann E.J., Wolin S.L. A role for a bacterial ortholog of the Ro autoantigen in starvation-induced rRNA degradation. Proc. Natl. Acad. Sci. U.S.A. 2010; 107:4022–4027. 20160119 10.1073/pnas.1000307107 PMC2840137

