# Supplementary material for: Polynucleotide phosphorylase promotes the stability and function of Hfq-binding sRNAs by degrading target mRNA-derived fragments
Source: Nucleic Acids Res. 2019 Jul 22;47(16):8821–37. doi: 10.1093/nar/gkz616 (PMC7145675; doi:10.1093/nar/gkz616)
Supplement: gkz616_Supplemental_Files [file gkz616_supplemental_files.zip › Cameron_Matz_De_Lay-supp_V2.pdf]

SUPPLEMENTAL INFORMATION

Polynucleotide phosphorylase promotes the stability and function of Hfq-binding sRNAs by degrading target mRNA-derived fragments.

Todd A. Cameron[1†], Lisa M. Matz[1†], Dhriti Sinha[1], and Nicholas R. De Lay[1,2,*]

[1] Department of Microbiology and Molecular Genetics, McGovern Medical School, The University of Texas Health Science Center, Houston, Texas, 77030, United States of America

[2] MD Anderson Cancer Center UTHealth Graduate School of Biomedical Sciences, The University of Texas Health Science Center, Houston, Texas, 77030, United States of America

* To whom correspondence should be addressed. Tel: 713-500-6293; Fax: 713-500-5499; Email: nicholas.r.delay@uth.tmc.edu

TABLE OF CONTENTS

Table S1. Strains and plasmids used in this study.

Table S2. Primers, probes, and gBlocks used in this study.

Figure S1. PNPase is critical for the decay of mRNA-derived fragments.

Figure S2. mRNA-derived fragments that accumulate in the absence of PNPase interact with Hfq.

Figure S3. The active site of PNPase is required for decay of mRNA-derived fragments and for stabilization of sRNAs, but not for RNA binding.

Figure S4. Substitutions in Hfq that block pairing of sRNAs with target mRNAs suppress the defect in sRNA levels that occurs in a Δ*pnp* strain.

Figure S5. Substitutions in Hfq residues critical for binding mRNA targets suppress the stability defect of sRNAs in a Δ*pnp* strain.

Figure S6. Mutations in stemloop 2 do not reduce the accelerated decay of RyhB that occurs in the absence of PNPase.

Figure S7. CyaR target-pairing drives its decay in the absence of PNPase.

Figure S8. Predicted structures of RNA fragments that accumulate in the Δ*pnp* strain.

Supplemental Materials and Methods

Supplemental References

Table S1. Strains and plasmids used in this study.

Strain	Relevant Features	Reference
MG1655	*rph-1*	(1)
KR10000	MG1655 rph^+	D. Court, NCI
CR201	*cyaR::kan ccdB*	C. Ranquet
DJS2820	*rph-1 ΔlacX74 mal::lacI*^{q} *ΔaraBAD hfqR17A*	(2)
DJS2864	*rph-1 ΔlacX74 mal::lacI*^{q} *ΔaraBAD* hfqY25D	(2)
EM1377	*rph-1 ΔlacX74 rne-131 zce-726::Tn10*	(3)
NRD463	*rph-1 Δpnp::cat*	(4)
NRD576	*rph-1 ΔlacX74 mal::lacI*^{q} *rne-131 zce-726::Tn10 Δpnp::kan*	(5)
NRD999	rph^+ *Δpnp::cat*	(5)
NRD1038λ	*rph-1 ΔryhB::kan-pBAD-ccdB* mini-λ_{tet}	(6)
NRD1138 (WT)	*rph-1*	(6)
NRD1139	*rph-1 Δpnp*	(6)
NRD1142	*rph-1 rne-131 zce-726::Tn10*	NRD1138 x P1(EM1377)
NRD1143	*rph-1 rne-131 zce-726::Tn10 Δpnp::cat*	NRD1139 x P1(EM1377)
NRD1243	rph^+ *pnp::pnp-3xFLAG*	(6)
NRD1410	*rph-1 hfqY25D*	DS057 x P1(DJS2864)
NRD1474	*hfq*R17A *Δpnp::kan*	DS060 x P1(NRD576)
NRD1478	*rph-1 hfqY25D Δpnp::kan*	NRD1410 x P1(NRD576)
NRD1597λ	*rph-1 ΔmgrR::ccdB kan*	DS091λ
NRD1599	*rph-1 ΔmgrR::mgrRmut*	NRD1597λ
NRD1601	*rph-1 ΔmgrR::mgrRmut Δpnp::cat*	NRD1599 x P1(NRD999)
DS021	*rph-1 Δhfq::kan*	(7)
DS057	*rph-1 Δhfq*::cat sacB *ΔpurA::kan*	(7)
DS060	*rph-1 hfqR17A*	(7)
DS070	*rph-1 Δpnp::kan*	NRD1138 x P1(NRD576)
DS091λ	*rph-1*	NRD1138 + mini-λ_{tet}
LM06	*rph-1 ryhB* G44C C45G	(7)
LM10	*rph-1 ΔmalQ ΔlacX*74 *ΔryhB*::*cat*	(7)
LM11 (*ryhBmut*)	*rph-1 ryhB* G44C C45G	(7)
LM12 (*Δpnp ryhBmut*)	*rph-1 ryhB* G44C C45G *Δpnp::cat*	LM11 x P1(NRD999)
LM23	*rph-1 ryhB* G53C C54G	NRD1038λ
LM24 (*ryhBmut2*)	*rph-1 ryhB* G53C C54G	LM10 x P1(LM23)
LM25 (*Δpnp ryhBmut2*)	*rph-1 ryhB* G53C C54G *Δpnp::cat*	LM24 x P1(NRD999)
LM26	*rph-1 ryhB* G44C C45G G53C C54G	NRD1038λ
LM27 (*ryhBdblmut*)	*rph-1 ryhB* G44C C45G G53C C54G	LM10 x P1(LM26)
LM28 (*Δpnp ryhBdblmut*)	*rph-1 ryhB* G44C C45G G53C C54G *Δpnp::cat*	LM27 x P1(NRD999)
TC222	rph^+*::*rph^+*-3xFLAG*	(6)
TC274	*Δrph::kan ΔcyaR ΔmicA::zeo(frt) Δpnp::cat*	(6)
TC292	*Δrph::kan ΔcyaR ΔmicA::zeo(frt)*	(6)

	ΔryhB::cyaR Δpnp::cat	
TC299	*Δrph::kan Δpnp::cat*	(6)
TC464	*rph-1 Δhfq::kan Δpnp::cat*	NRD1139 x P1(DS021)
TC465λ	*rph-1 cyaR::ccdB kan*	DS091λ
TC468 (*cyaRmut*)	*rph-1 cyaR* CC42GG CC45GG	TC465λ
TC476 (Δ*pnp cyaRmut*)	*rph-1 cyaR* CC42GG CC45GG Δ*pnp::cat*	TC468 x P1(NRD1137)
**Plasmid**	**Relevant Features**	**Reference**
pCA24N	*lacI*^{q}	(8)
pBRplac	Amp^R; P_{lac}	(9)
pTC329	Amp^R; P_{lac} *araB-5'UTR pnp3xFLAG*	pBRplac
pTC331	Amp^R; P_{lac} *araB-5'UTR rph3xFLAG*	pBRplac
pTC352	Amp^R; P_{lac} *araB-5'UTR pnp3xFLAG lacI*^{q}	pTC329
pTC353	Amp^R; P_{lac} *araB-5'UTR rph3xFLAG lacI*^{q}	pTC331
pTC354	Amp^R; P_{lac} *araB-5'UTR pnp(S438A) 3xFLAG lacI*^{q}	pTC352
pTC356	Amp^R; P_{lac} *araB-5'UTR pnp(D492G) 3xFLAG lacI*^{q}	pTC352
pTC396	Amp^R; P_{lac} *lacI*^{q}	pTC353
pTC402	Amp^R; P_{lac} *araB-5'UTR pnp(S437-9A)3xFLAG lacI*^{q}	pTC352

Table S2. Primers, probes, and gBlocks used in this study.

**Primers**	**Sequence (5' – 3')**
PNP Forward	GGGACGTCGGTTAGGGTTGTCATTAGTCG
PNP Reverse	GGGACGTCGAATGAACGTCCTGTTCCC
RyhBmut2	GCGATCAGGAAGACCCTCGCGGAGAACCTGAAAGCACGACATTGCTCACATTCGTTCCAGTATTACTTAGCCAGCCGGGTGCTGGCTTTTTTTTT
RyhBdblmut	GCGATCAGGAAGACCCTCGCGGAGAACCTGAAAGCACGACATTCGTCACATTCGTTCCAGTATTACTTAGCCAGCCGGGTGCTGGCTTTTTTTTT
RyhB Overlap PCR Forward	CAAGTGCGAATGAGAATGATTATTATTGTCTCGCGATCAGGAAGACC
RyhB Overlap PCR Reverse	GCACTCCCGTGGATAAATTGAGAACGAAAGATCAAAAAAAAAGCCGC
RyhBKOchk Forward	CAAATGCGAGTCAAATGC
RyhBKOchk Reverse	GTGGTTCCTACTGGAGTGC
MgrRccdBKan For	GAAAATGCCTGTTAGCGTAAAAGCAAAACACAAATCTATCTTATATTCCCCAGAACATCAGG
MgrRccdBkan Rev	GGCGAAAAAAAACCGCCAGTAAACCGGCGGTGAATGCTTGATAGGAACTTCAAGATCC
MgrRmut2	TGCCTGTTAGCGTAAAAGCAAAACACAAATCTATCGTACCAAGCATTCACCGCCGGTTTACTGGCGGTTTTTTTTCG
MgrRKOchk For	GCTACTGTGTCAACGATTCG
MgrRKOchk Rev	GAACGGAGATAATCCCTCACC
cyaR ccdB up	AAGCTATGCCGATAGCACCAGGCGATGGTTATACTGTGTGATTATATTCCCCAGAACATCAGG
cyaR ccdB down	AAAAATAAGCCCGTGTAAGGGAGATTACACAGGCTAAGGAATAGGAACTTCAAGATCCCC
cyaR fwd	AAGCTATGCCGATAGCACCAGGCGATGGTTATACTGTGTGGCTCCCGGTCCCGCAGGATC

cyaR 4G rvs	AAAAATAAGCCCGTGTAAGGGAGATTACACAGGCTAAGGACCTCCTTCC TGGTACAGCTAGCATT
cyaR check fwd	GCACCAGGCGATGGTTATAC
cyaR check rvs	ATGTGTGGACGTGACCAGAA
pnp_BAD-5f-AatII	ATTGCGACGTCCAAACCCGTTTTTTTGGATGGAGTACTAGTTTGCTTAAT CCGATCGTTCGTAAATTCC
rph_BAD-5f-AatII	ATTGCGACGTCCAAACCCGTTTTTTTGGATGGAGTACTAGTATGCGTCCA GCAGGCCGTAGCAATA
3xf_term-3r-EcoRI	GCAATGAATTCAAAAAAAAAACCCCGCCCTGTCAGGGGCGGGGTTTTTT TTTTTATTTATCGTCGTCATCTTTGTAG
lacIq HindIII fwd	ATTGCAAGCTTTAATGCGGTAGTGCAAAACCTTTCGCGGTATGG
lacIq StyI rvs	GCAATCCAAGGTCACTGCCCGCTTTCCAGTCG
pnp S438A fwd	CACGGAAGCCATAGAAGCGGAACCGTTGGATTCAG
pnp S438A rvs	CTGAATCCAACGGTTCCGCTTCTATGGCTTCCGTG
pnp D492G fwd	ACCTGCAACTTTGAAGCCCATATCGCCCAGGTG
pnp D492G rvs	CACCTGGGCGATATGGGCTTCAAAGTTGCAGGT
pnp S437-9A fwd	GCACACGGAAGCCATAGCAGCGGCACCGTTGGATTCAGTGA
pnp S437-9A rvs	TCACTGAATCCAACGGTGCCGCTGCTATGGCTTCCGTGTGC
**Probes**	
RyhB-LM	[Btn]GTCGTGCTTTCAGGTTCTCCGCGAGGGTCTTCC
RyhB	[Btn]AAGTAATACTGGAAGCAATGTGAGCAATGTCGTGCTTTCAGGTTCTC
CyaR	[Btn]TGGTTCCTGGTACAGCTAGCATTTTATGGGTTATG
GcvB	[Btn]CCAGAACACGCATTCCGATAAAACTTTTCGTTCCGGCTCAGG
McaS	[Btn]AGCAGTGCATCCGCGTCTTAAATCC
MgrR	[Btn]CAGTAAACCGGCGGTGAATGCTTGCATGGATAGAT
MgrR2	[Btn]CGCTAACAGGCATTTTCCTGCACTGATAACGAATC
ArcZ	[Btn]GGCTAGACCGGGGTGCGCGAATACTGCGCCAACACCAGGG
ChiX	[Btn]CATTTTTTTATTATTATGCCGTCACTTTAAGCGACGGTG
SsrA	[Btn]CGCCACTAACAAACTAGCCTGATTAAGTTTTAACGCTTCA
cfa.5UTR	[Btn]AGGAACCCTTGCCGAAATGACTATCCATAGTGAGT
cfaprobe1	[Btn]CCACCAGCCATCCATATAACTTTCGCCTAACCCCA
chiP	[Btn]GTTAATCCTCTTTGACGTATAAATTGCTGCACCAAAGGTG
dsbB	[Btn]CACTGCGGCATTTCCAGACCTAAAAAATCCCACTG
hemN.5UTR	[Btn]CTTCCGATGGCGTCTCTGGCGGCTACGGCGTACCG
miaA.3UTR	[Btn]AAAAAATTGCGCACGATACGTCTCAATTGTACACA
ompX_probe1	[Btn]TACGGAAGTACCTGCGGTGAAAGCCAGAACTGCGG
yqaE.RBS	[Btn]GATTCTCCAGAAACCCATATGTACTCCCTATAAGA
**gBlock**	
RyhBmut	GTGTTGGACAAGTGCGAATGAGAATGATTATTATTGTCTCGCGATCAGG AAGACCCTCGCGGAGAACCTGAAAGCACGACATTCGTCACATTGCTTCC AGTATTACTTAGCCAGCCGGGTGCTGGCTTTTTTTTTGATCTTTCGTTCT CAATTTATCCACGGGAGTGCTTGTGTT

SUPPLEMENTAL FIGURES

Figure S1

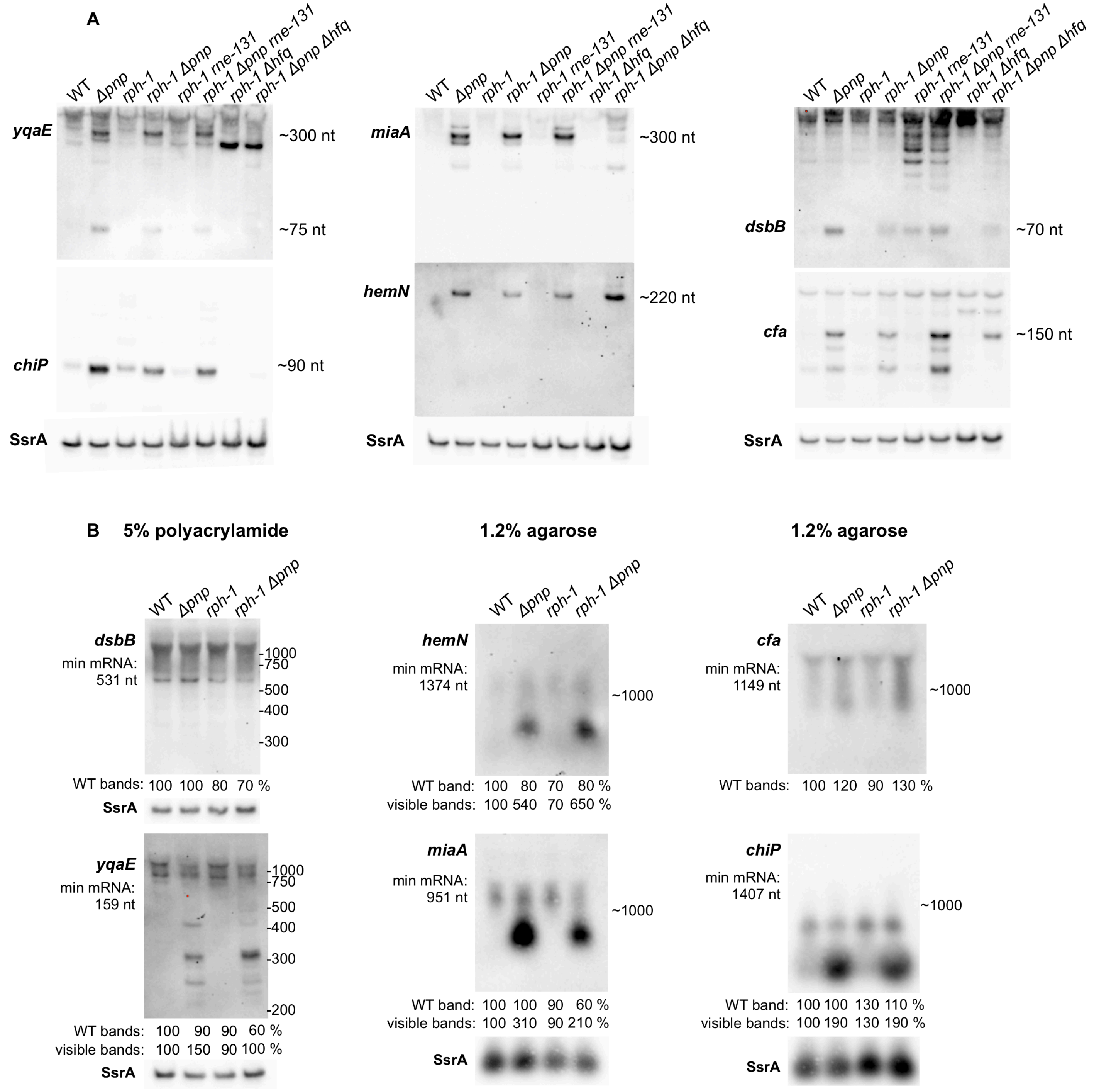

Figure S1. PNPase is critical for the decay of mRNA-derived fragments. (**A**) Expanded view of the northern blots shown in Fig 1D for wild-type (KR10000), Δ*pnp* (NRD999), *rph-1* (NRD1138), and *rph-1* Δ*pnp* (NRD1139), plus additional lanes for *rph-1 rne-131* (NRD1142), *rph-1 rne-131* Δ*pnp* (NRD1143), *rph-1 Δhfq* (DS021), and *rph-1 Δhfq* Δ*pnp* (TC464) strains grown to OD_{600} 1.0. Blots were cropped to

show all relevant bands observed with each probe. Gene names and approximate band sizes are shown adjacent to bands displayed in the main figure. (**B**) Northern blots of larger RNA transcripts associated with the mRNA fragments. Numbers under each lane indicate the average relative intensity of three replicates for the bands present in the wild type lane or for all the bands visible in each lane, as indicated. SsrA served as a loading control. All blots except for *cfa* were probed with the same probes used to detect the small mRNA fragment; *cfa* utilized a different mRNA-specific probe (cfaprobe1). RNA ladder sizes are marked on the right of each blot.

Figure S2

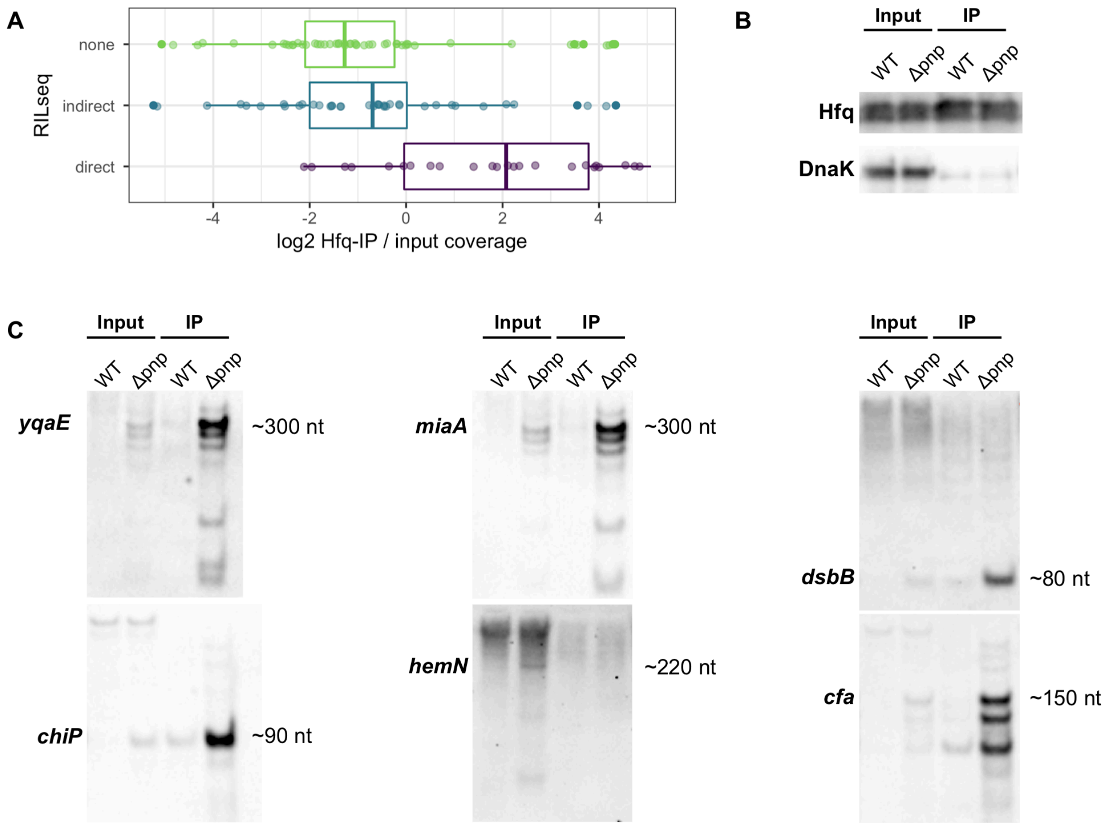

Figure S2. mRNA-derived fragments that accumulate in the absence of PNPase interact with Hfq. (**A**) Boxplots showing the $\log_2$ fold change in normalized coverage for fragments in the Hfq co-IP fraction versus the input fraction of the Δ*pnp* strain (NRD999). Fragments were categorized depending on the presence of a direct coordinate overlap with a RIL-seq sRNA-pairing region hit, an indirect overlap with a RIL-seq sRNA-pairing region hit to the same gene, or no RIL-seq sRNA-pairing region hits. (**B**) Hfq protein levels in input and IP fractions detected by western blot. DnaK levels are shown as a control. Blots are representative of three replicates. (**C**) Expanded view of the northern blots shown in Fig 2C. Blots were cropped to show all relevant bands observed with each probe. Gene names and approximate band sizes are shown adjacent to bands displayed in the main figure.

Figure S3

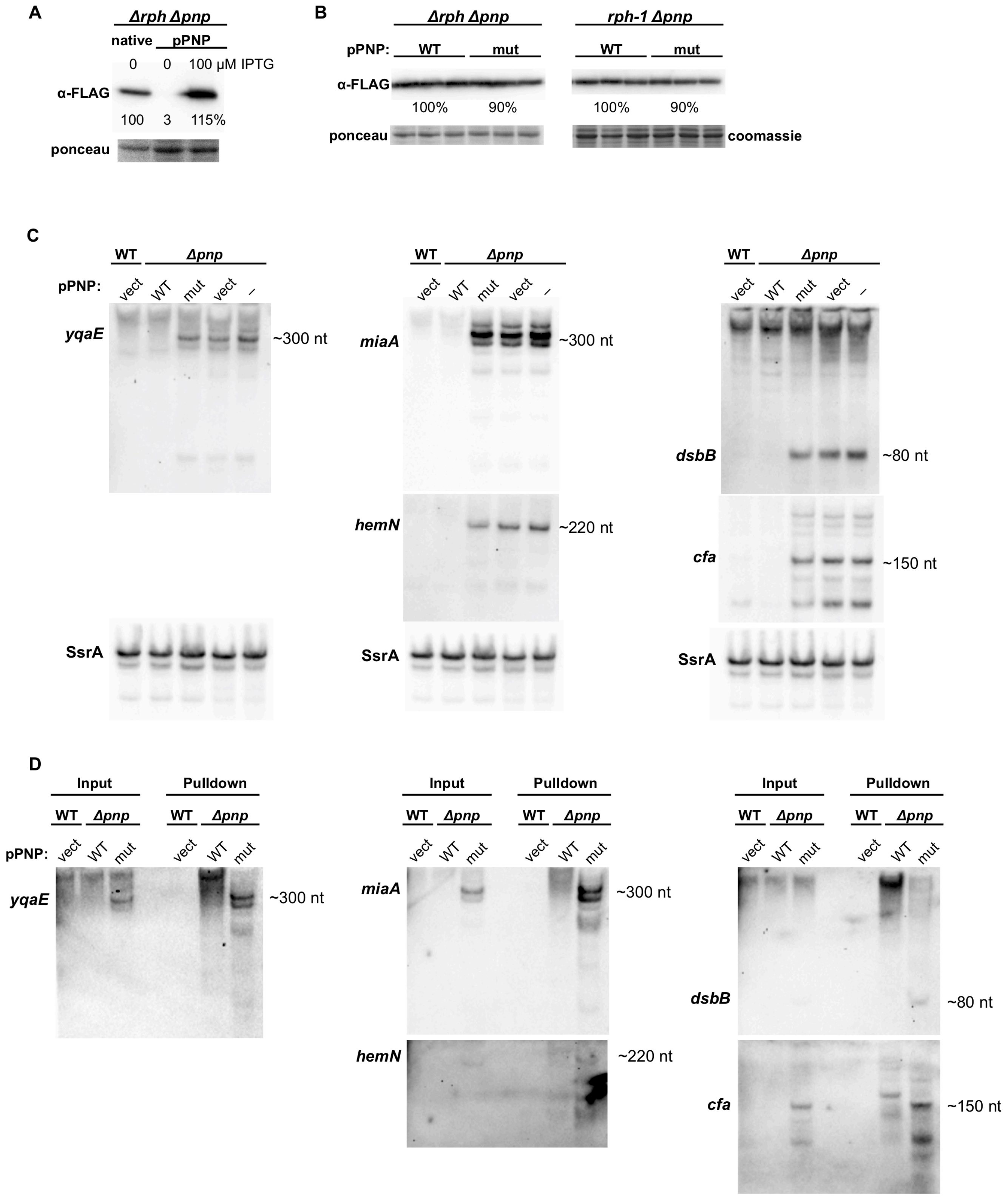
A
Δrph Δpnp
native
pPNP
0
0
100 μM IPTG
α-FLAG
100
3
115%
ponceau
B
Δrph Δpnp
rph-1 Δpnp
pPNP:
WT
mut
WT
mut
α-FLAG
100%
90%
100%
90%
ponceau
coomassie
C
WT
Δpnp
pPNP:
vect
WT
mut
vect
–
yqaE
~300 nt
miaA
~300 nt
hemN
~220 nt
dsbB
~80 nt
cfa
~150 nt
SsrA
D
Input
Pulldown
WT
Δpnp
pPNP:
vect
WT
mut
yqaE
~300 nt
miaA
~300 nt
hemN
~220 nt
dsbB
~80 nt
cfa
~150 nt

Figure S3, cont.

Supplemental Figure S3., cont.

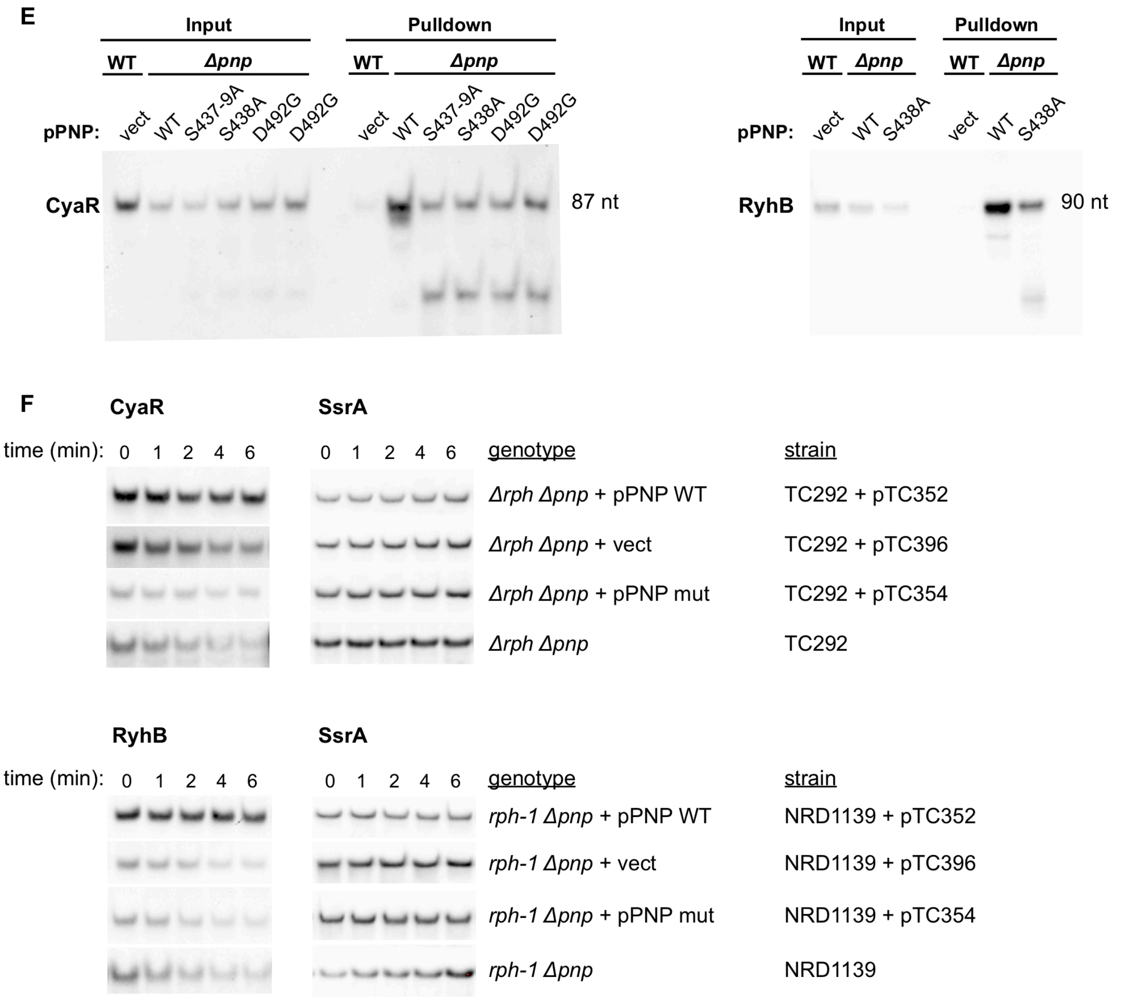

Figure S3. The active site of PNPase is required for decay of mRNA-derived fragments and for stabilization of sRNAs, but not for RNA binding. (**A**) Protein expression levels of PNPase-3xFLAG wild-type (WT) expressed from the native locus (NRD1243) or from a plasmid vector in a Δ*rph* Δ*pnp* (TC274) strain grown without induction or grown in the presence of 100 µM IPTG. Numbers indicate the average expression of three replicates relative to the natively-expressed protein. Total protein visualized by Ponceau served as a loading control. (**B**) Protein expression levels of the PNPase-3xFLAG wild-type (WT) and S438A catalytic mutant (mut) expressed from a plasmid vector in both the Δ*rph* Δ*pnp* (TC299) and *rph-1* Δ*pnp* (NRD1138) strain backgrounds. Numbers indicate the average relative expression of three replicates. Blots were probed with α-FLAG antibody. Total protein visualized by Ponceau or by a Coomassie-stained replicate gel served as a loading control. (**C**) and (**D**) Expanded view of the northern blots shown in Fig 3A and B. Blots were cropped to show all relevant bands observed with each probe. Gene names and approximated band size are shown next to the bands displayed in the main figure. (**C**) Expression of fragments in the input fractions of PNPase-3xFLAG pulldowns. (**D**) PNPase-3xFLAG

pulldown of RNA fragments. Input fractions were loaded at ~1:190 dilutions relative to IP fractions. (**E**) Input and PNPase-3xFLAG pulldown fractions probed for the presence of the sRNAs CyaR and RyhB in a WT (KR10000) strain containing the vector control (vect) or in a Δ*pnp* (NRD999) strain containing pPNP expressing either the PNPase-3x FLAG wild-type (WT) or the indicated PNPase catalytic mutant. Samples were collected at OD_{600} of 1.0. RyhB samples were collected after treating cultures with dipyridyl for 15 min. Input fractions were loaded at ~1:190 dilutions relative to IP fractions. (**F**) Representative blots of CyaR and RyhB stability time courses corresponding to Fig 3C and D.

Figure S4

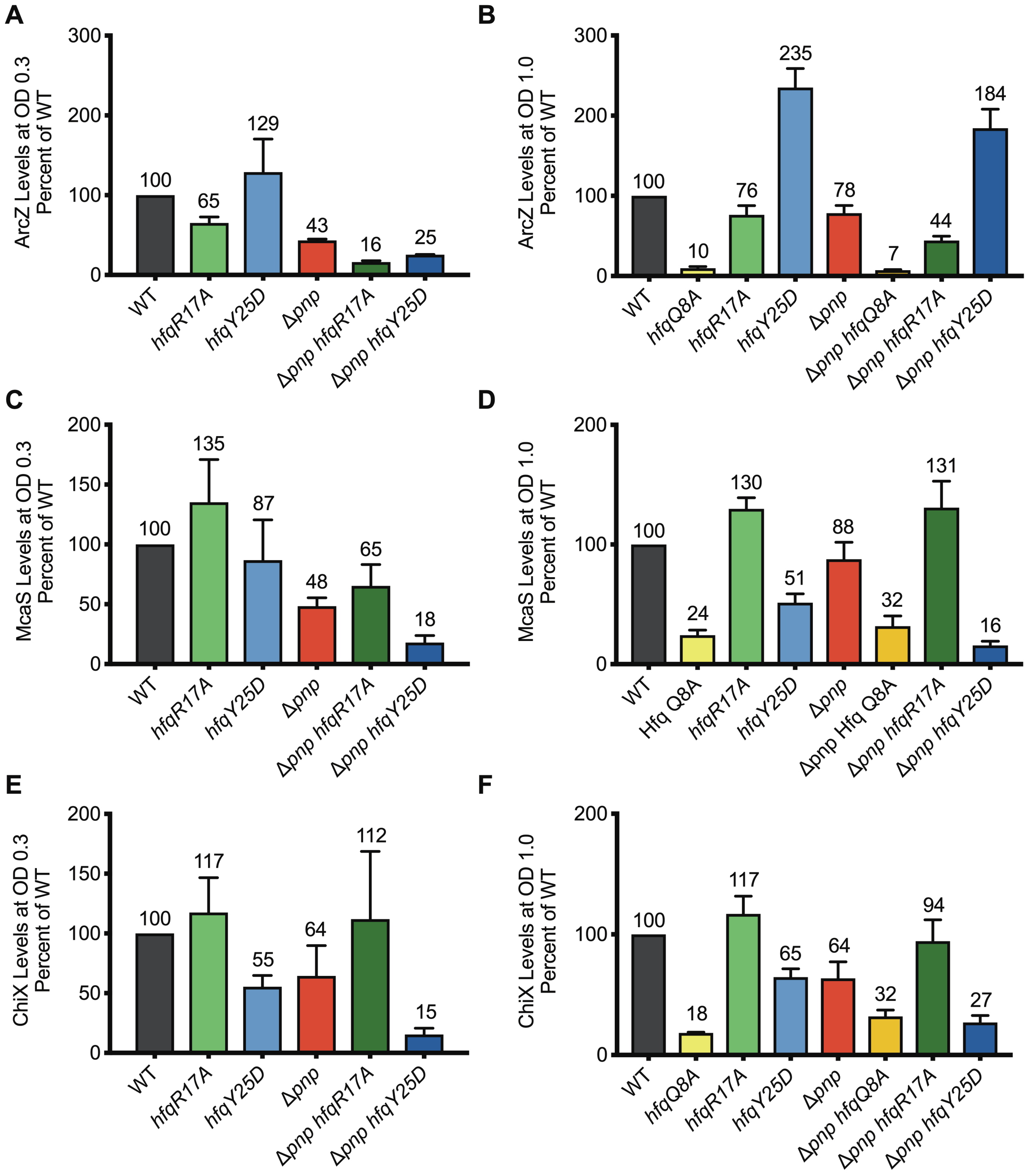

Figure S4. Substitutions in Hfq that block pairing of sRNAs to target mRNAs suppress the defect in sRNA levels that occurs in a Δ*pnp* strain. ArcZ (**A**, **B**), McaS (**C**, **D**), and ChiX levels (**E**, **F**) were determined for strains NRD1138 (WT), DS070 (Δ*pnp*), DS058 (*hfqQ8A*) DS060 (*hfqR17A*), NRD1410 (*hfqY25D*), NRD1473 (Δ*pnp hfqQ8A*), NRD1474 (Δ*pnp hfqR17A*), and NRD1478 (Δ*pnp hfqY25D*) as detailed in Figure 4. Bars indicate the mean expression from three independent experiments for each sRNA. sRNA levels were normalized to the control RNA SsrA and scaled to WT. Error bars represent the standard error of the mean. ArcZ is a Class I sRNA, while McaS and ChiX are Class II sRNAs.

Figure S5

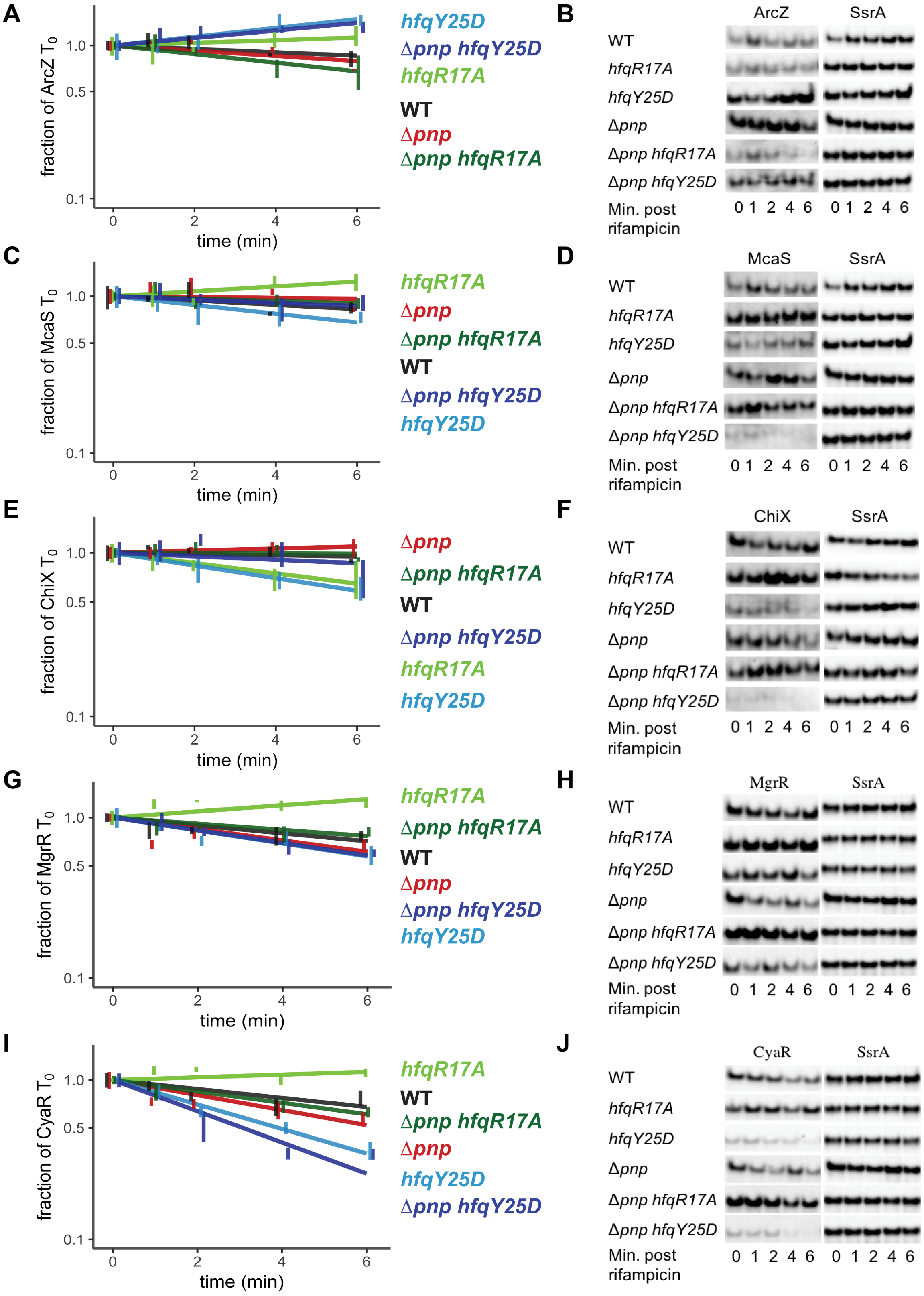
A
fraction of ArcZ T_0
time (min)
hfqY25D
Δpnp hfqY25D
hfqR17A
WT
Δpnp
Δpnp hfqR17A
B
ArcZ
SsrA
WT
hfqR17A
hfqY25D
Δpnp
Δpnp hfqR17A
Δpnp hfqY25D
Min. post rifampicin
0 1 2 4 6
0 1 2 4 6
C
fraction of McaS T_0
time (min)
hfqR17A
Δpnp
Δpnp hfqR17A
WT
Δpnp hfqY25D
hfqY25D
D
McaS
SsrA
WT
hfqR17A
hfqY25D
Δpnp
Δpnp hfqR17A
Δpnp hfqY25D
Min. post rifampicin
0 1 2 4 6
0 1 2 4 6
E
fraction of ChiX T_0
time (min)
Δpnp
Δpnp hfqR17A
WT
Δpnp hfqY25D
hfqR17A
hfqY25D
F
ChiX
SsrA
WT
hfqR17A
hfqY25D
Δpnp
Δpnp hfqR17A
Δpnp hfqY25D
Min. post rifampicin
0 1 2 4 6
0 1 2 4 6
G
fraction of MgrR T_0
time (min)
hfqR17A
Δpnp hfqR17A
WT
Δpnp
Δpnp hfqY25D
hfqY25D
H
MgrR
SsrA
WT
hfqR17A
hfqY25D
Δpnp
Δpnp hfqR17A
Δpnp hfqY25D
Min. post rifampicin
0 1 2 4 6
0 1 2 4 6
I
fraction of CyaR T_0
time (min)
hfqR17A
WT
Δpnp hfqR17A
Δpnp
hfqY25D
Δpnp hfqY25D
J
CyaR
SsrA
WT
hfqR17A
hfqY25D
Δpnp
Δpnp hfqR17A
Δpnp hfqY25D
Min. post rifampicin
0 1 2 4 6
0 1 2 4 6

Figure S5. Substitutions in Hfq residues critical for binding mRNA targets suppress the stability defect of sRNAs in a Δ*pnp* strain. ArcZ (**A**, **B**), McaS (**C**, **D**), ChiX (**E**, **F**), MgrR (**G, H**), and CyaR (**I, J**) stability was measured in early exponential phase (OD_{600} of 0.3 for ArcZ, McaS, and ChiX) or late exponential phase (OD_{600} of 1.0 for MgrR and CyaR) cultures of strains NRD1138 (WT), DS070 (Δ*pnp*), DS060 (*hfqR17A*), NRD1410 (*hfqY25D*), NRD1474 (Δ*pnp hfqR17A*), and NRD1478 (Δ*pnp hfqY25D*) as detailed in Figure 5. ArcZ and McaS levels were measured by northern blot analysis using RNA samples from the CyaR induction experiment, and ChiX and MgrR levels were measured by northern blot analysis using RNA samples from the RyhB induction experiment described in Figure 5. sRNA levels were normalized to the control RNA SsrA. Graphs represent the mean from three independent experiments for each sRNA, and error bars represent the standard error of the mean.

Figure S6

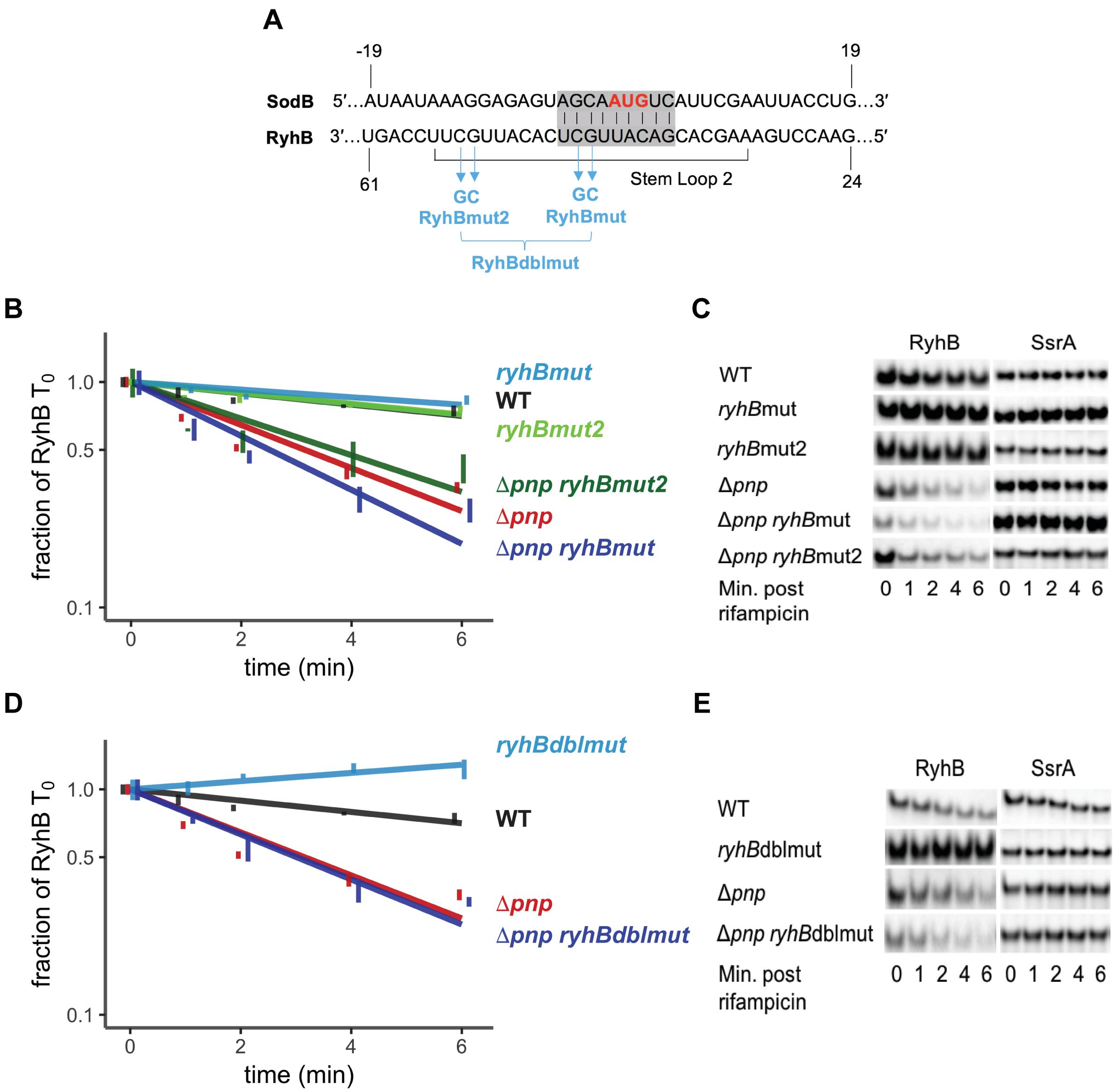

Figure S6. Mutations in stemloop 2 do not reduce the accelerated decay of RyhB that occurs in the absence of PNPase. (**A**) Illustration showing complementarity between RyhB and *sodB* (highlighted in grey) and three mutations (blue) that were introduced into *ryhB* (*ryhBmut, ryhBmut2,* and *ryhBdblmut*). The start codon of *sodB* is depicted in red. (**B, D**) Stability curves of the single (**B**) or double (**D**) RyhB mutants in WT and Δ*pnp* backgrounds. Overnight cultures of strain NRD1138 (WT), a *pnp* deletion strain (Δ*pnp*, DS070), or derived strains harboring mutations in *ryhB* (*ryhBmut*, LM11; Δ*pnp ryhBmut*, LM12; *ryhBmut2*, LM24; Δ*pnp ryhBmut2*, LM25; *ryhBdblmut*, LM27; Δ*pnp ryhBdblmut*, LM28) were diluted into fresh LB medium and grown to early exponential phase (OD_{600} of 0.3). RyhB was induced for 15 min by the addition of 2, 2' dipyridyl. Rifampicin RNA stability time courses were performed as described for Figure 3. RyhB levels were normalized to the control RNA SsrA and graphed as a fraction of initial RyhB

level (T_0). Results represent the mean of at least three independent experiments and the error bars represent the standard error of the mean. (**C, E**) Representative blots for each sRNA.

Figure S7

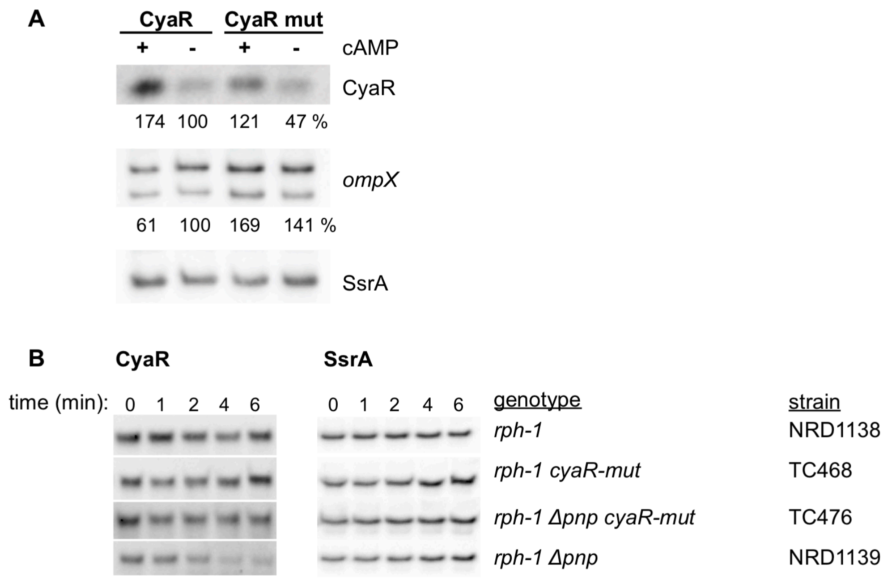

Figure S7. CyaR target-pairing drives its decay in the absence of PNPase. (**A**) Representative blots of CyaR and *ompX* levels corresponding to Fig 7B. Numbers under each band indicate the average of three replicates and are relative to the averages in the CyaR wild type or pairing mutant strains grown without cAMP induction. (**B**) Representative blots of CyaR stability time courses corresponding to Fig 7C. SsrA served as a loading control.

Figure S8

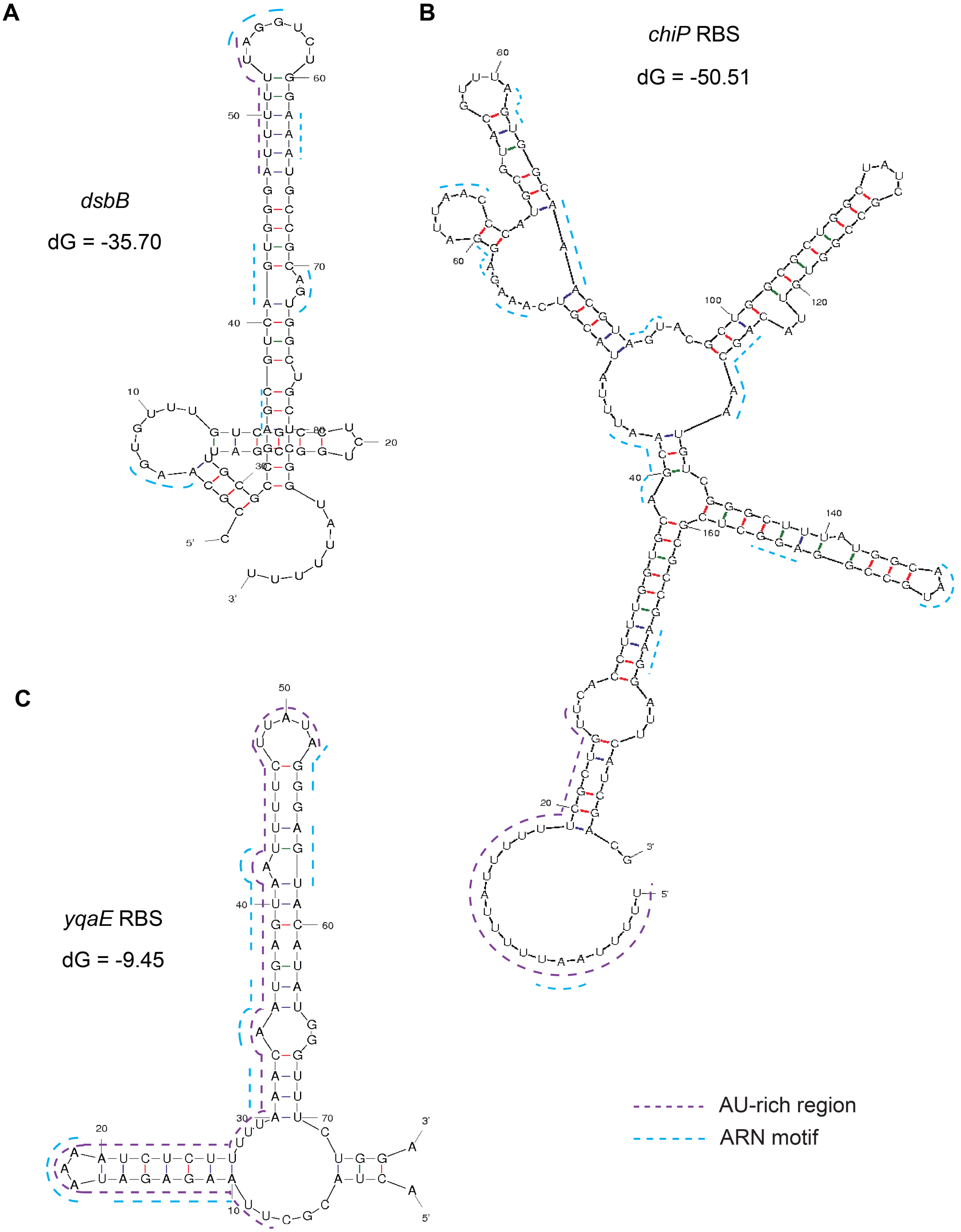

A
dsbB
dG = -35.70
B
chiP RBS
dG = -50.51
C
yqaE RBS
dG = -9.45
AU-rich region
ARN motif

Figure S8, cont.

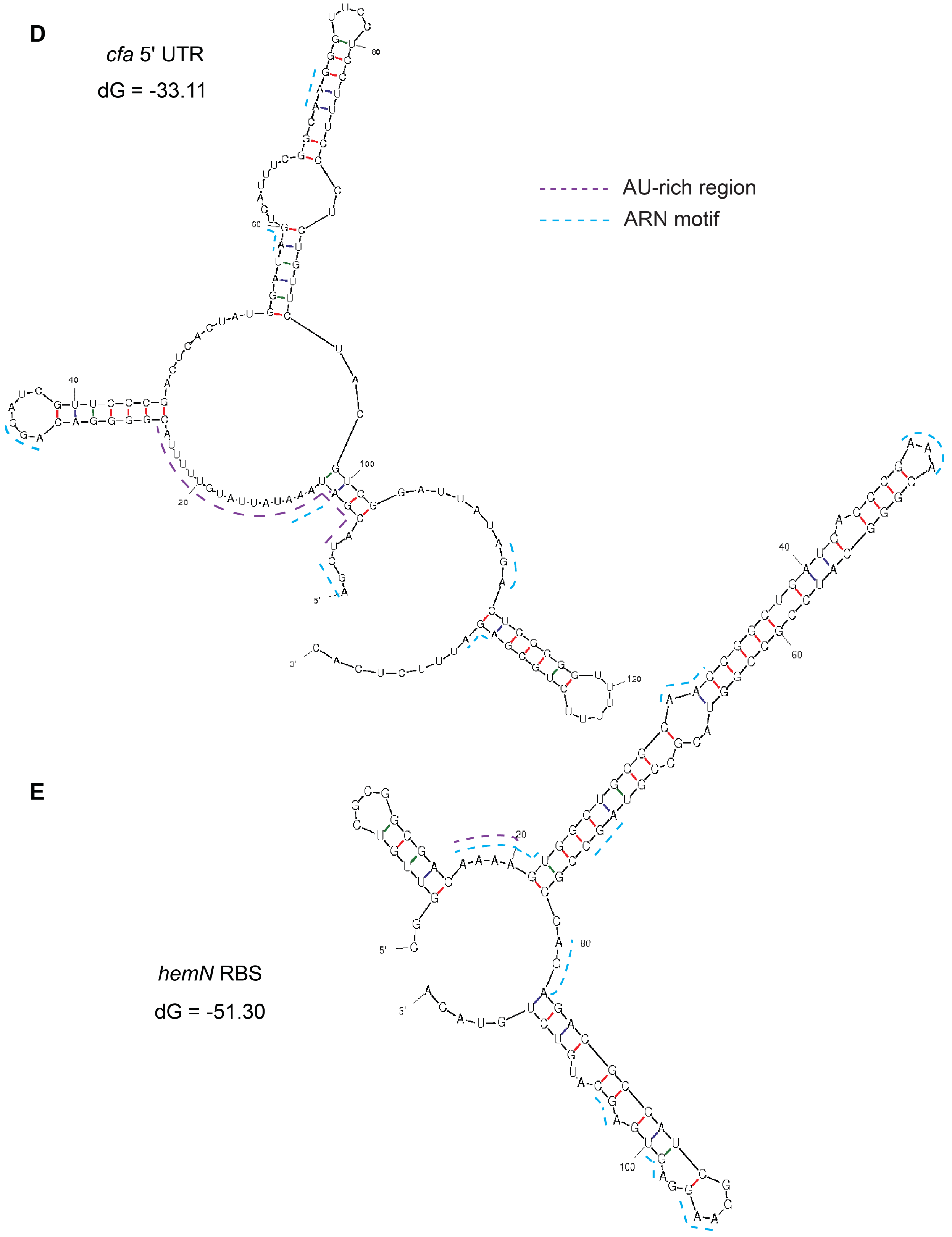

Figure S8, cont.

F

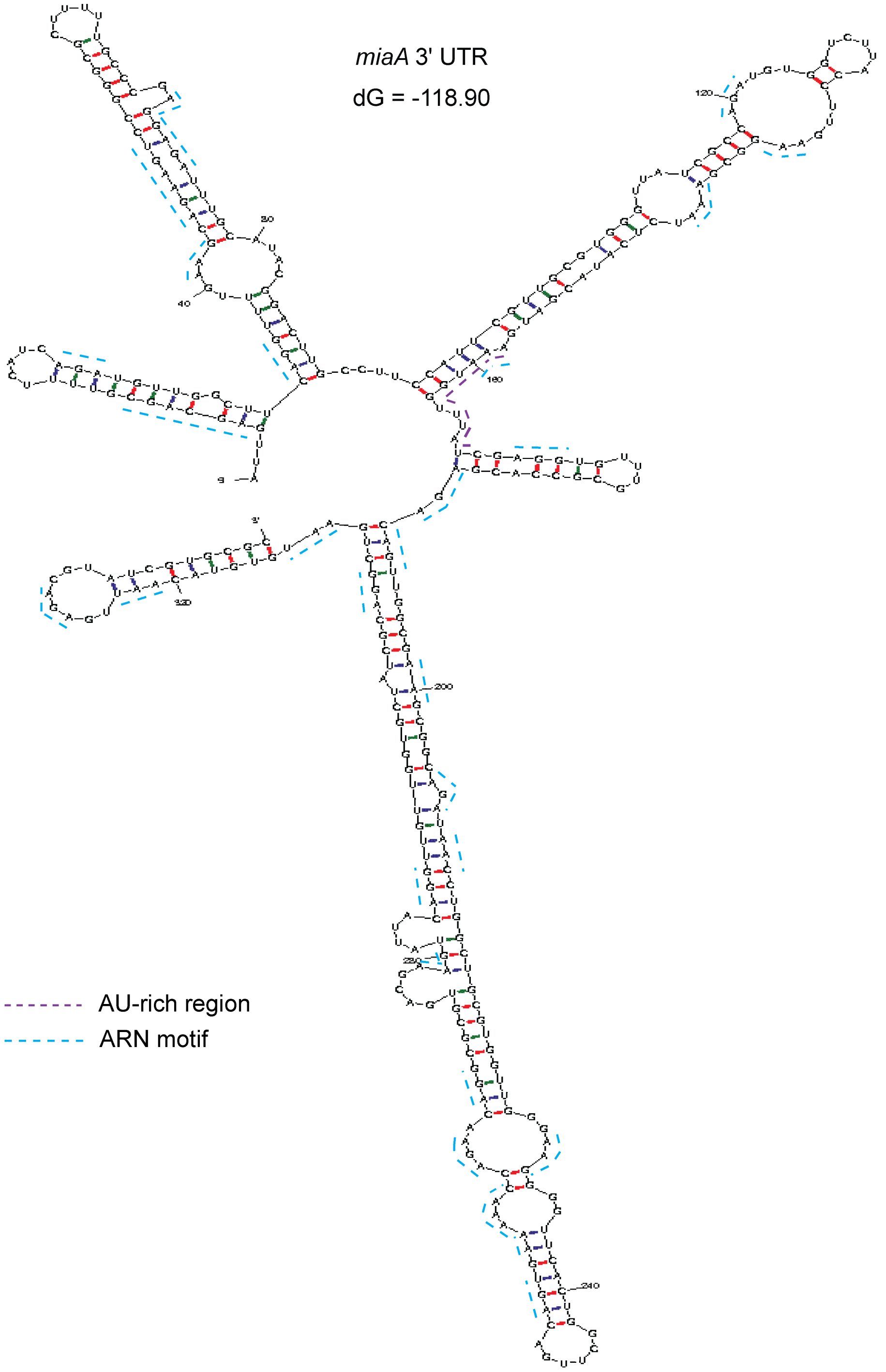

Figure S8. Predicted structures of RNA fragments that accumulate in the Δ*pnp* strain. Minimum-free energy structures are shown for mRNA fragments detected by short RNA-seq and associated with *dsbB* (**A**), *chiP* (**B**), *yqaE* (**C**), *cfa* (**D**), *hemN* (**E**), and *miaA* (**F**) using Mfold (http://unafold.rna.albany.edu). ΔG (kcal/mol) values displayed beneath structure names. Predicted ARN motifs and AU-rich regions are indicated by blue and purple dashed lines, respectively.

SUPPLEMENTAL MATERIALS AND METHODS

Hfq immunoprecipitation.

Input protein for western blot analysis was taken from initial whole-cell lysates. Protein from IP samples was precipitated from the initial phenol fraction of the RNA extraction using 2 volumes of cold acetone and pelleted at 18,000 x g for 30 min. The pellet was washed twice with 1 volume of cold acetone, pelleted at 18,000 x g for 10 min after each wash, and was allowed to air dry. The protein was then suspended in Laemmli sample buffer.

Western blots

Western blots were performed as described previously (7). Hfq was detected using 1:5000 dilution of pre-absorbed anti-Hfq antiserum and goat anti-rabbit IgG secondary antibody (Thermo Fisher). For detection of DnaK (loading control) 1:10,000 dilution of mouse anti-DnaK monoclonal antibody (Abcam) and anti-mouse goat secondary antibody (Santa Cruz Biotechnologies, Inc) were used following manufacturer's guidelines. 3xFLAG-tagged proteins were detected using 1:10,000 rat anti-FLAG antibody (Millipore Sigma) and 1:2500 goat anti-rat antibody (Stratagene). All secondary antibodies were conjugated to alkaline phosphatase and were visualized using Immun-Star^{TM} AP substrate (Bio-Rad).

Strain and plasmid construction

Strains generated by P1*vir* transduction using the protocol described by Miller (10) are indicated in Supplementary Table S1 with the donor strain indicated in parentheses. Strains bearing the "λ" suffix were previously transformed with purified mini-λ_{tet} for use with lambda Red-mediated recombination (11). Final lambda Red recombinants and plasmid constructs were verified by sequencing. Primers and synthesized DNA fragments (gBlocks) were purchased from Integrated DNA Technologies (Coralville, IA) and from MilliporeSigma (St. Louis, MO). Restriction enzymes and T4 DNA ligase were purchased from New England Biolabs (Ipswich, MA).

<u>NRD1597λ:</u> A PCR product was first generated using the primers *MgrRccdBKan For* and *MgrRccdBKan Rev* to amplify a Kan-pBAD-*ccdB* cassette from strain CR201 with flanking homology to replace *mgrR*. Strain NRD1138λ was transformed with this PCR product, and tet^R kan^R arabinose^S recombinants were subsequently isolated and verified by colony PCR using the primers *MgrRKOchk For* and *MgrRKOchk Rev*.

<u>NRD1599:</u> Strain <u>NRD1597λ</u> was transformed with the *MgrRmut2* oligo (Table S2) in order to replace the Kan-pBAD-*ccdB* cassette with a mutated version of *mgrR*. Recombinants that were kan^S and arabinose^R were isolated and verified by sequencing using the primers *cyaR check fwd* and *cyaR check rvs*.

<u>LM06:</u> NRD1038λ harboring mini-lambda phage and the Kan-pBAD-*ccdB* cassette was transformed with the *RyhBmut* gBlock (IDT, Table S2) containing the mutated *ryhB* sequence with 40 bp of homology on either side of *ryhB*. Successful recombinants (growth on 0.2% arabinose, kan^S) were sequence-verified after colony PCR amplification using *RyhBKOchk* forward and reverse primers.

<u>LM23 and LM26:</u> ssOligos (Sigma, Table S2) containing a single GC→CG inversion at positions 53 and 54 (RyhBmut2), or a double inversion at positions 44 and 45, and 53 and 54 (RyhBdblmut) were amplified by overlap extension PCR using the *RyhB Overlap* PCR forward and reverse primers. The PCR products were then transformed into strain NRD1038λ harboring mini-lambda phage and the Kan-pBAD-*ccdB* cassette. Successful recombinants were sequence-verified using *RyhBKOchk* forward and reverse primers.

<u>TC465λ:</u> A PCR product was first generated using the primers *cyaR ccdB up* and *cyaR ccdB down* to amplify a Kan-pBAD-*ccdB* cassette from strain CR201 with flanking homology to replace *cyaR*. Strain DS091λ was transformed with this PCR product, and tet^R kan^R arabinose^S recombinants were subsequently isolated and verified by sequencing using the primers *cyaR check fwd* and *cyaR check rvs*.

<u>TC468:</u> Strain TC465λ was transformed with the PCR product generated from the primers *cyaR fwd* and *cyaR 4G rvs* in order to replace the Kan-pBAD-*ccdB* cassette with a mutated version of *cyaR*.

Recombinants that were kan^S and $arabinose^R$ were isolated and verified by sequencing using the primers *cyaR check fwd* and *cyaR check rvs*.

pTC329 and pTC331: Insert DNA for pTC329 was amplified using primers *pnp_BAD-5f-AatI* and *3xf_term-3r-EcoRI* with genomic DNA from strain NRD1243 carrying *pnp-3xFLAG*. pBRplac vector DNA and PCR insert DNA were digested with AatI and EcoRI then ligated together. pTC331 was constructed identically, except that the primers *rph_BAD-5f-AatI* and *3xf_term-3r-EcoRI* were used with genomic DNA from strain TC222 carrying *rph-3xFLAG.*

pTC352 and pTC353: $LacI^q$ was amplified from pCA24N using primers *lacIq HindIII fwd* and *lacIq StyI rvs*. pTC329 or pTC331 vector DNA and PCR insert DNA were digested with HindIII and StyI then ligated together.

pTC354, pTC356, pTC402: Mutations were introduced into *pnp* on the pTC352 plasmid using site-directed mutagenesis with the primers *pnp S438A fwd* and *pnp S438A rvs* (pTC354), *pnp D492G fwd* and *pnp D492G rvs* (pTC356), and *pnp S437-9A fwd* and *pnp S437-9A rvs* (pTC402).

pTC396: pTC353 DNA was digested with EcoRI and AatII to excise *rph-3xFLAG*, blunted with Mung Bean Nuclease, then re-ligated to obtain the empty vector.

SUPPLEMENTAL REFERENCES

1. Blattner, F.R., Plunkett, G., 3rd, Bloch, C.A., Perna, N.T., Burland, V., Riley, M., Collado-Vides, J., Glasner, J.D., Rode, C.K., Mayhew, G.F. *et al.* (1997) The complete genome sequence of *Escherichia coli* K-12. *Science*, **277**, 1453-1462.
2. Schu, D.J., Zhang, A., Gottesman, S. and Storz, G. (2015) Alternative Hfq-sRNA interaction modes dictate alternative mRNA recognition. *EMBO J*, **34**, 2557-2573.
3. Masse, E., Escorcia, F.E. and Gottesman, S. (2003) Coupled degradation of a small regulatory RNA and its mRNA targets in *Escherichia coli*. *Genes Dev*, **17**, 2374-2383.
4. De Lay, N. and Gottesman, S. (2011) Role of polynucleotide phosphorylase in sRNA function in *Escherichia coli*. *RNA*, **17**, 1172-1189.
5. Bandyra, K.J., Sinha, D., Syrjanen, J., Luisi, B.F. and De Lay, N.R. (2016) The ribonuclease polynucleotide phosphorylase can interact with small regulatory RNAs in both protective and degradative modes. *RNA*, **22**, 360-372.
6. Cameron, T.A. and De Lay, N.R. (2016) The Phosphorolytic Exoribonucleases Polynucleotide Phosphorylase and RNase PH Stabilize sRNAs and Facilitate Regulation of Their mRNA Targets. *J Bacteriol*, **198**, 3309-3317.
7. Sinha, D., Matz, L.M., Cameron, T.A. and De Lay, N.R. (2018) Poly(A) polymerase is required for RyhB sRNA stability and function in *Escherichia coli*. *RNA*, **24**, 1496-1511.
8. Kitagawa, M., Ara, T., Arifuzzaman, M., Ioka-Nakamichi, T., Inamoto, E., Toyonaga, H. and Mori, H. (2005) Complete set of ORF clones of *Escherichia coli* ASKA library (a complete set of *E. coli* K-12 ORF archive): unique resources for biological research. *DNA Res*, **12**, 291-299.
9. Guillier, M. and Gottesman, S. (2006) Remodelling of the *Escherichia coli* outer membrane by two small regulatory RNAs. *Mol Microbiol*, **59**, 231-247.
10. Miller, J.H. (1992) *A short course in bacterial genetics : a laboratory manual and handbook for Escherichia coli and related bacteria*. Cold Spring Harbor Laboratory Press, Plainview, N.Y.
11. Yu, D., Ellis, H.M., Lee, E.C., Jenkins, N.A., Copeland, N.G. and Court, D.L. (2000) An efficient recombination system for chromosome engineering in *Escherichia coli*. *Proc Natl Acad Sci U S A*, **97**, 5978-5983.
